# Clinical applications of neurofeedback based on sensorimotor rhythm: a systematic review and meta-analysis

**DOI:** 10.3389/fnins.2023.1195066

**Published:** 2023-11-20

**Authors:** Tatiana Ferri Ribeiro, Marcelo Alves Carriello, Eugenio Pereira de Paula, Amanda Carvalho Garcia, Guilherme Luiz da Rocha, Helio Afonso Ghizoni Teive

**Affiliations:** ^1^Internal Medicine and Health Sciences, Federal University of Paraná (UFPR), Curitiba, Paraná, Brazil; ^2^Physical Education (UFPR)—Invited Colaborador, Federal University of Paraná (UFPR), Curitiba, Paraná, Brazil; ^3^Department of Clinical Medicine, UFPR, and Coordinator of the Movement Disorders Sector, Neurology Service, Clinic Hospital, Federal University of Paraná (UFPR), Curitiba, Paraná, Brazil

**Keywords:** sensory motor rhythm, pathology, standard therapy, neurologia, neuropsiquiatría

## Abstract

**Background:**

Among the brain-machine interfaces, neurofeedback is a non-invasive technique that uses sensorimotor rhythm (SMR) as a clinical intervention protocol. This study aimed to investigate the clinical applications of SMR neurofeedback to understand its clinical effectiveness in different pathologies or symptoms.

**Methods:**

A systematic review study with meta-analysis of the clinical applications of EEG-based SMR neurofeedback performed using pre-selected publication databases. A qualitative analysis of these studies was performed using the Consensus tool on the Reporting and Experimental Design of Neurofeedback studies (CRED-nf). The Meta-analysis of clinical efficacy was carried out using Review Manager software, version 5.4.1 (RevMan 5; Cochrane Collaboration, Oxford, UK).

**Results:**

The qualitative analysis includes 44 studies, of which only 27 studies had some kind of control condition, five studies were double-blinded, and only three reported a blind follow-up throughout the intervention. The meta-analysis included a total sample of 203 individuals between stroke and fibromyalgia. Studies on multiple sclerosis, insomnia, quadriplegia, paraplegia, and mild cognitive impairment were excluded due to the absence of a control group or results based only on post-intervention scales. Statistical analysis indicated that stroke patients did not benefit from neurofeedback interventions when compared to other therapies (Std. mean. dif. 0.31, 95% CI 0.03–0.60, *p* = 0.03), and there was no significant heterogeneity among stroke studies, classified as moderate *I*^2^ = 46% *p*-value = 0.06. Patients diagnosed with fibromyalgia showed, by means of quantitative analysis, a better benefit for the group that used neurofeedback (Std. mean. dif. −0.73, 95% CI −1.22 to −0.24, *p* = 0.001). Thus, on performing the pooled analysis between conditions, no significant differences were observed between the neurofeedback intervention and standard therapy (0.05, CI 95%, −0.20 to −0.30, *p* = 0.69), with the presence of substantial heterogeneity *I*^2^ = 92.2%, *p*-value < 0.001.

**Conclusion:**

We conclude that although neurofeedback based on electrophysiological patterns of SMR contemplates the interest of numerous researchers and the existence of research that presents promising results, it is currently not possible to point out the clinical benefits of the technique as a form of clinical intervention. Therefore, it is necessary to develop more robust studies with a greater sample of a more rigorous methodology to understand the benefits that the technique can provide to the population.

## 1 Introduction

New treatment approaches based on neuroimaging techniques are being evaluated to promote clinical improvements in neurological/neuropsychiatric diseases in cases of patients resistant to conventional treatment such as occupational therapy, physiotherapy, or psychotherapy (Sitaram et al., 2017). Brain-machine interfaces, which aim to provide real-time feedback to the patient during interventions, are expanding, with neurofeedback being the most common (Dias, 2010).

Neurofeedback uses operant conditioning as a working model (Sitaram et al., 2017). By performing a cognitive task to modulate the brain regions and/or rhythms of interest, the patients receive real-time feedback on their brain activity (Sitaram et al., 2017), becoming able to optimize such cognitive strategies to achieve the desired neuromodulation. This technique can be based on different neuroimaging modalities, but due to portability, lower costs, and practicality issues, and its non-invasive character, currently, the electroencephalogram (EEG) is the most used (Buch et al., 2012; Sitaram et al., 2017).

Different EEG rhythms can be directed during neurofeedback training, sensorimotor rhythm (SMR) being one of the most common targets, which allows for continuous and asynchronous control (Edelman et al., 2014). The capture of cerebral signals can occur through different modalities, with the three main types of capture being signals based on the electroencephalogram (EEG), in which electrodes are placed under the scalp, through cortical surface electrocorticography (ECOG), and in the brain through action potentials through a single neuron using single units, in addition to extraneural potentials in cortical layers, which are placed at different field distances and spatial resolutions (Edelman et al., 2014).

Such sensor-based intervention processes make use of event-related phenomena that are observed at specific electrodes placed along the motor cortex, these neurons maintain an idle firing rate in the alpha/mu band (8–13 Hz) and perform synchronization within focal regions based on the type of task being performed (Edelman et al., 2014). When a certain movement is executed/imagined, the sensor-base intervention action encodes the cortical processes of neurons for different movements, interrupting the idle state resulting in the process of desynchronization of certain local populations of neurons (Neuper et al., 2006; Edelman et al., 2014).

This action acts by interrupting the idle state, resulting in the desynchronization of local populations, such a phenomenon is called event-related synchronization (ERS) and event-related desynchronization (ERD; Neuper et al., 2006). The preparation process, execution, and imagination of the movement produces the process ERD, in sensory-motor areas, in alpha and beta bands, and the mu ERD band is more present in contralateral sensorimotor areas during motor preparation and extends bilaterally with the beginning of the movement (Neuper et al., 2006; Rimbert and Fleck, 2023).

In this way, motor intention can be decoded from the SMR and act as responsible for generating neural control in neurofeedback (Yuan and He, 2014), and its application in different pathologies, stroke (Schabus et al., 2017), fibromyalgia, in healthy individuals aiming to achieve high performance is being investigated (Dias, 2010; Spychala et al., 2020; Veikko et al., 2021).

Although there is a growing number of neurofeedback studies in the literature, the level of evidence is still questionable, as the exact mechanisms of SMR modulation are still unknown (Edelman et al., 2014). Many studies weren't adequately designed, without control groups or randomization, have biased reporting of their results, and the experimental setting is also variable between studies, with high variability in the number and location of channels, frequency ranges, feedback modality, between others (Sorger et al., 2019; Ros et al., 2020) (Table 1). Thus, this study performed a systematic review and meta-analysis of the clinical applications of EEG-based SMR neurofeedback, with the objective of understanding the most investigated pathologies, experimental designs, and the clinical efficacy reached by this potential intervention.

**Table 1:** Presentation of articles included in the meta-analysis.

**Reference**	**Pathology**	**Study design**	**Training dose**	**Sessions (N)**	**Scale**
Rayegani et al., 2014	stroke	RCT	10 OT sessions (5 sessions per week for 2 weeks, 60 min); TONF and TOBF groups received neurofeedback or EMG-BF therapies at the end of each occupational therapy session 3x a week for 4 weeks	10	Jebsen Test (JHFT)
Chen et al., 2020	stroke	RCT	10 OT sessions (5 sessions per week for 2 weeks, 60 min); TONF and TOBF groups received neurofeedback or EMG-BF therapies at the end of each occupational therapy session 3x a week for 4 weeks	12	FMA
Li et al., 2014	stroke	RCT	3x a week 1 to 1h and 5 min session for 8 weeks	24	FMA
Pichiorri et al., 2015	stroke	RCT	1 month of training:	12	FMA
Wang et al., 2018	stroke	RCT	3-5 x per week completed between 5-7 weeks	20	FMA
Remsik et al., 2019	stroke	Cross Over	2 to 3 sessions per week 2 hours each	9-15	ARAT
Wu et al., 2020	stroke	RCT	4 weeks 5 days a week 2h GC and 1h GE	20	FMA
Miao et al., 2020	stroke	RCT	3 x a week for 4 weeks	12	FMA
Mrachacz-Kersting et al., 2016	stroke	RCT	NI	3	FMA
Kayiran et al., 2010	FMG	RCT	24 week intervention NFB vs Escitalopram 10mg	20	VAS-pain
Wu et al., 2021	FMG	RCT	8 weeks	20	FIQR

## 2 Methodology

This review followed the guidelines of the Preferred Reporting Items for Systematic Reviews and Meta-Analysis (PRISMA) guidelines (Liberati et al., 2009).

### 2.1 Systematic search and selection of studies

**Figure 1:**
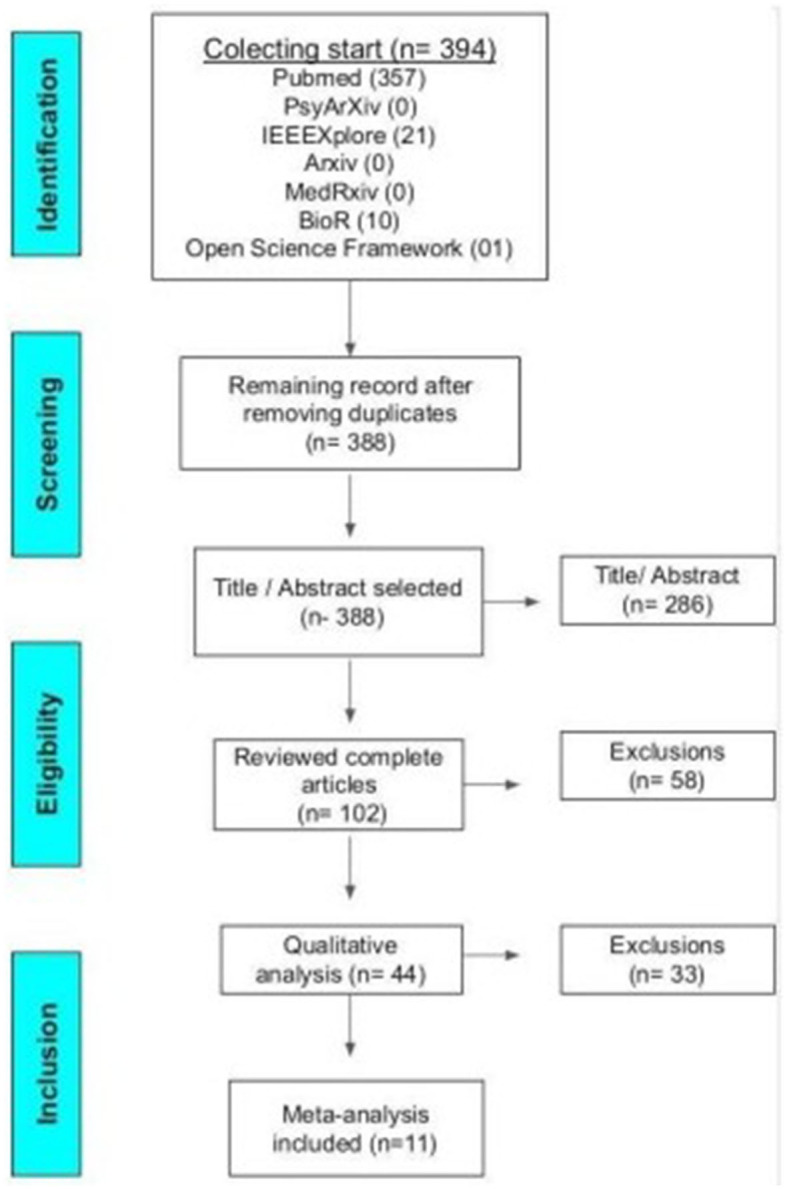
PRISMA flow diagram of the systematic review.

During the months of October, 2021 to January 25th, 2022, the analyses of the titles and abstracts of the articles were carried out. During the months of January 26th to May 30th, 2022, the articles were read in full. From May to July, a qualitative analysis was carried out. The research was performed using Pubmed, PsyArXiv, IEEEXplore, bioArxiv, MedRxiv, and Open Science Framework bibliographic databases and all found articles are represented in Figure 1. Were analyzed studies between the years 1995 and 2021. This review followed the Preferred Reporting Items for Systematic Reviews and Meta-Analysis (PRISMA) guidelines (Liberati et al., 2009).

The search in the databases was based on the following logical phrase: (neurofeedback OR “brain-computer interface”) AND [sensorimotor OR (sensory AND motor) OR SMR] AND (patient^*^ OR clinic^*^).

Three hundred and ninety-four articles were found initially, after excluding duplicates, 388 articles were selected and analyzed based on the inclusion and exclusion criteria as shown in Table 2 below.

**Table 2:** Inclusion and exclusion.

**Inclusion criteria**
1. Studies that present original results in adult humans (>21 years)
2. Studies including patients with a formal clinical diagnosis of neurological, motor, or psychiatric disorders; studies published in English
**Exclusion criteria**
1. Studies evaluating only healthy participants
2. Studies applying biofeedback based only on non-neural signals
3. Studies without voluntary control of brain activity
4. Studies with animal models
5. Articles with invasive procedures
6. Studies that do not use EEG
7. Case report (*n* < 5)
8. Articles, comments, editorials, or purely methodological protocols
9. Conference papers

To collect important information, a data extraction list was prepared, which included 45 items that were extracted by the coauthors (T.F.R and E.P.P.J) and the information was grouped into: study information (title, year, journal, author, objective), population (number of subjects in the experimental group, number of subjects in the control group, age, gender, education, disease, inclusion criteria, exclusion criteria, comorbidity, scale of symptoms, medical treatments used or other interventions, symptoms of severity), experimental design (controlled, randomized, blinded study, existence of follow-up), task (procedure, experimental group task, control group task, experimental group instructions, control group instructions, description of the experimental group session, description of the control group session, number of sessions), hardware/reprocessing (neuroimaging technique, imaging hardware/software, position of channels, number of channels, frequencies used, data processing, processing software), primary outcomes (clinical outcomes scale-based, descriptive clinical outcomes, non-clinical outcomes, outcomes, number of dropouts and reasons), outcomes during follow-up (follow-up, how many patients completed the observed outcomes at follow-up).

### 2.2 Evaluation of the experimental design and quality of the report used

The analysis of the quality of the study was performed using the Consensus on the Reporting and Experimental Design of Clinical and Cognitive-Behavioral Neurofeedback Studies (CRED-nf Checklist; Ros et al., 2020). This checklist is divided into essential and suggested items related to pre-experimental recording, control groups and measures, feedback specifications, description of results and data storage (Ros et al., 2020). Three coauthors (T.F.R; M.A.C; E.P.P.J) independently assessed the studies included in this review, which was based on the 23 criteria of the CRED-nf checklist. Disagreements between reviewing co-authors were resolved through discussion meetings.

### 2.3 Evaluation of the experimental design and quality of the report used

The statistical analysis was carried out using Review Manager software, version 5.4.1 (RevMan 5; Cochrane Collaboration, Oxford, UK). Low *I*^2^ heterogeneity was considered for <25%; ≤ 50% moderate; >75% substantial. To perform the forest plot, some scales were converted to be in the same direction, taking the result of the average obtained in the study of the maximum value of the scale. The standardized mean difference and the fixed effect were used for the analysis.

**Figure 2:**
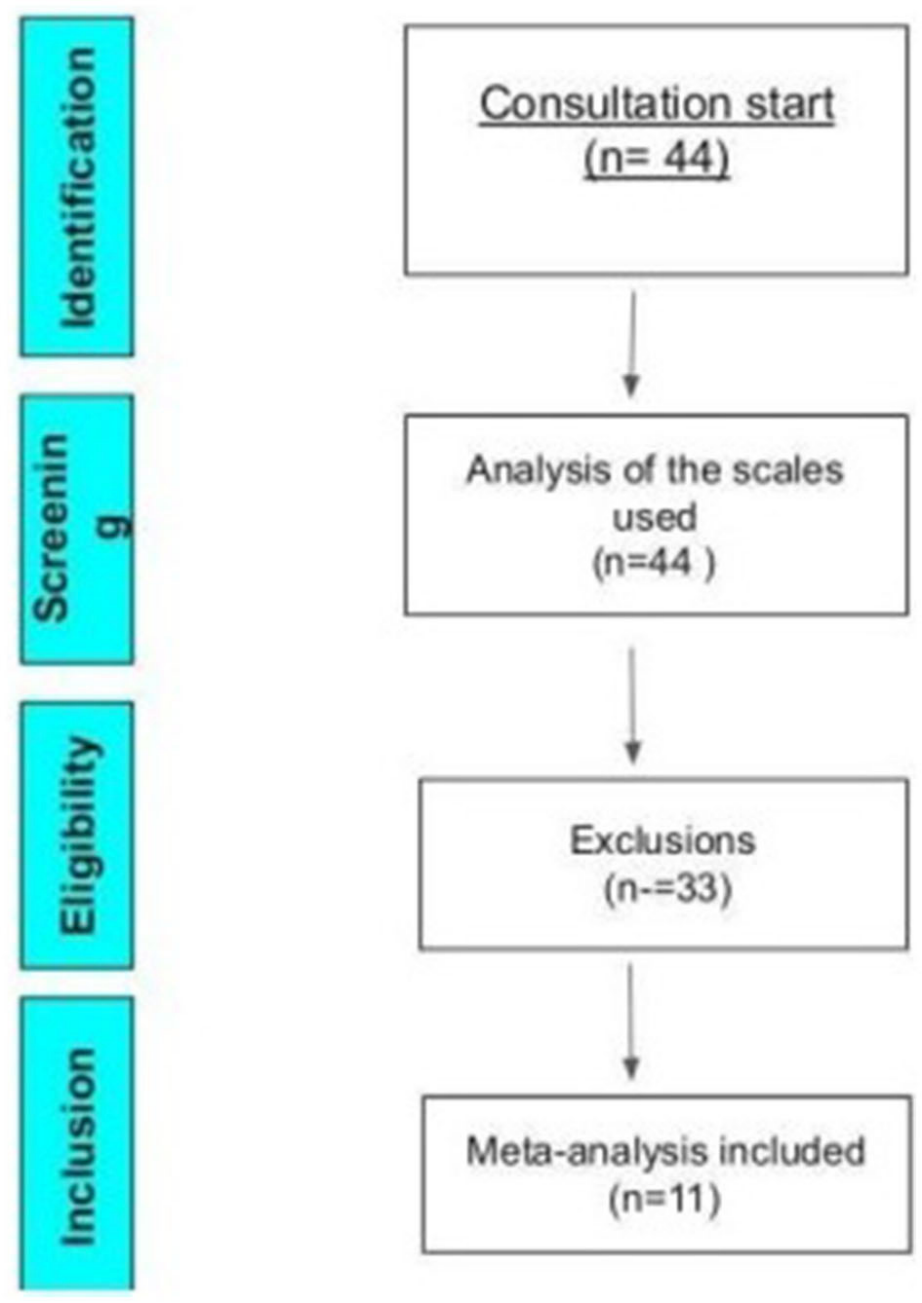
PRISMA flow diagram of the meta-analysis.

To be included in this meta-analysis, studies should be randomized or cross-over, with a control group, including pre- and post-intervention assessment scales with their results presented in tables, graphs, or the body of the text. The studies that did not show clinical improvements in percentages, but that provided enough data to allow the calculation of the percentage, were converted and standardized using the Excel platform. A total of 11 studies were included in the meta-analysis, as represented by Figure 2 of the Prism.

## 3 Results

### 3.1 Experimental variability of selected studies

The qualitative analysis indicated the characteristics of the studies (Supplementary Table 1), in which they were composed of protocols that, although using the same electrophysiological pattern (SMR), presented differences in the number of participants between the control and experimental groups, which varied from no control group to passive controls, containing participants present on a waiting list or interventions based on telephone contact (Wu et al., 2021).

**Figure 3:**
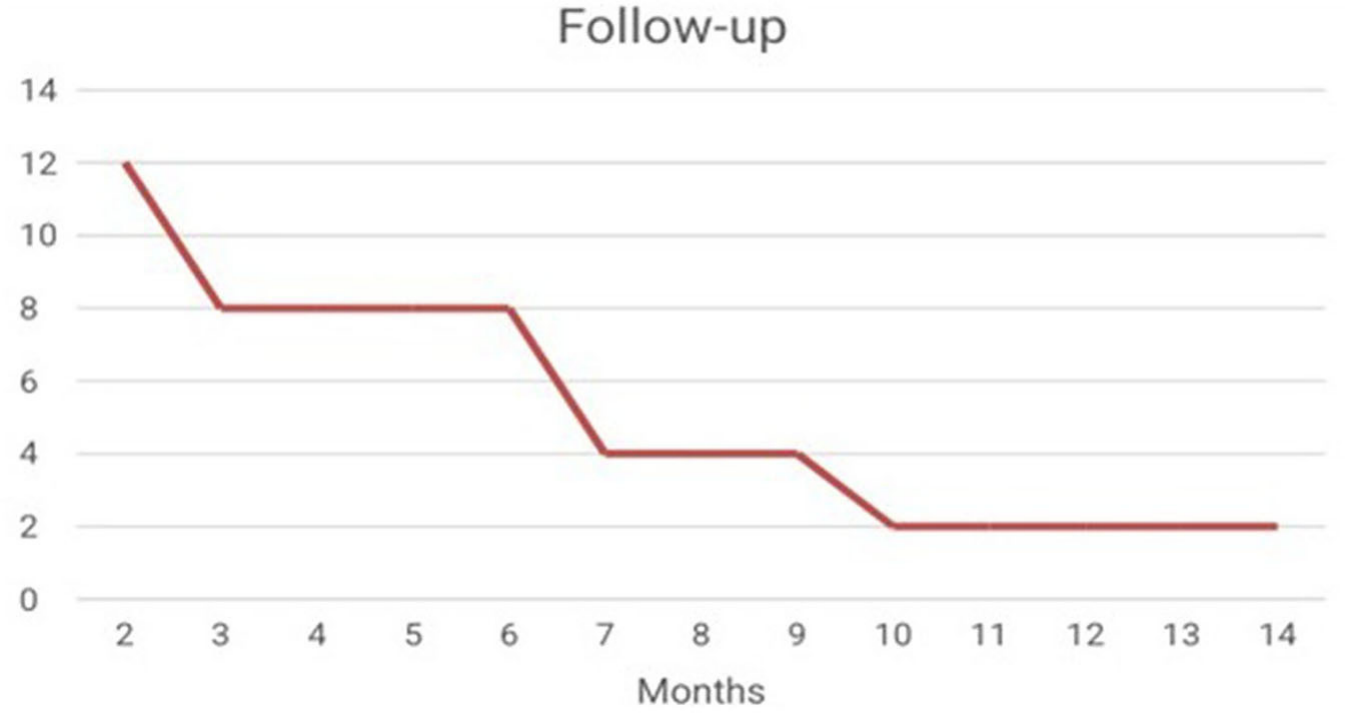
Sum of the number of articles that performed follow-up after intervention.

It was possible to verify the presence of the use of neuroimaging techniques such as fMRI, as an auxiliary way of evaluating the results (Shindo et al., 2011; Pinter et al., 2021). With regard to the presence of Follow-up, it is noted that after the interventions of the 44 studies analyzed, only 12 studies carried out the follow-up (Figure 3) (Hammer et al., 2011; Shindo et al., 2011; Schabus et al., 2017; Marlats et al., 2020; Lau et al., 2021).

The predominant type of feedback among stroke studies refers to the use of motor imagination during training, followed by auditory, visual, and exoskeleton feedback. A study of spinal cord injury, amputation of lower limbs, tetraplegia, and paraplegia also used motor imagination as feedback. However, studies of insomnia, Attention Deficit Hyperactivity Disorder (ADHD), Fibromyalgia, as well as two studies of Quadriplegia, Paraplegia, and Multiple Sclerosis, used visual/auditory feedback.

With regard to the type of feedback and the activity performed by the patient throughout the sessions, the results pointed to the presence of visual, tactile, and auditory feedback. It was possible to see that studies on insomnia, ADHD, Fibromyalgia, as well as two studies on Quadriplegia Paraplegia and Multiple Sclerosis, used visual/auditory feedback. However, the instructions provided during training referred to being oriented to move the mouse cursor, or images on the computer screen, or even imagine that they were moving the paralyzed or injured limb.

**Figure 4:**
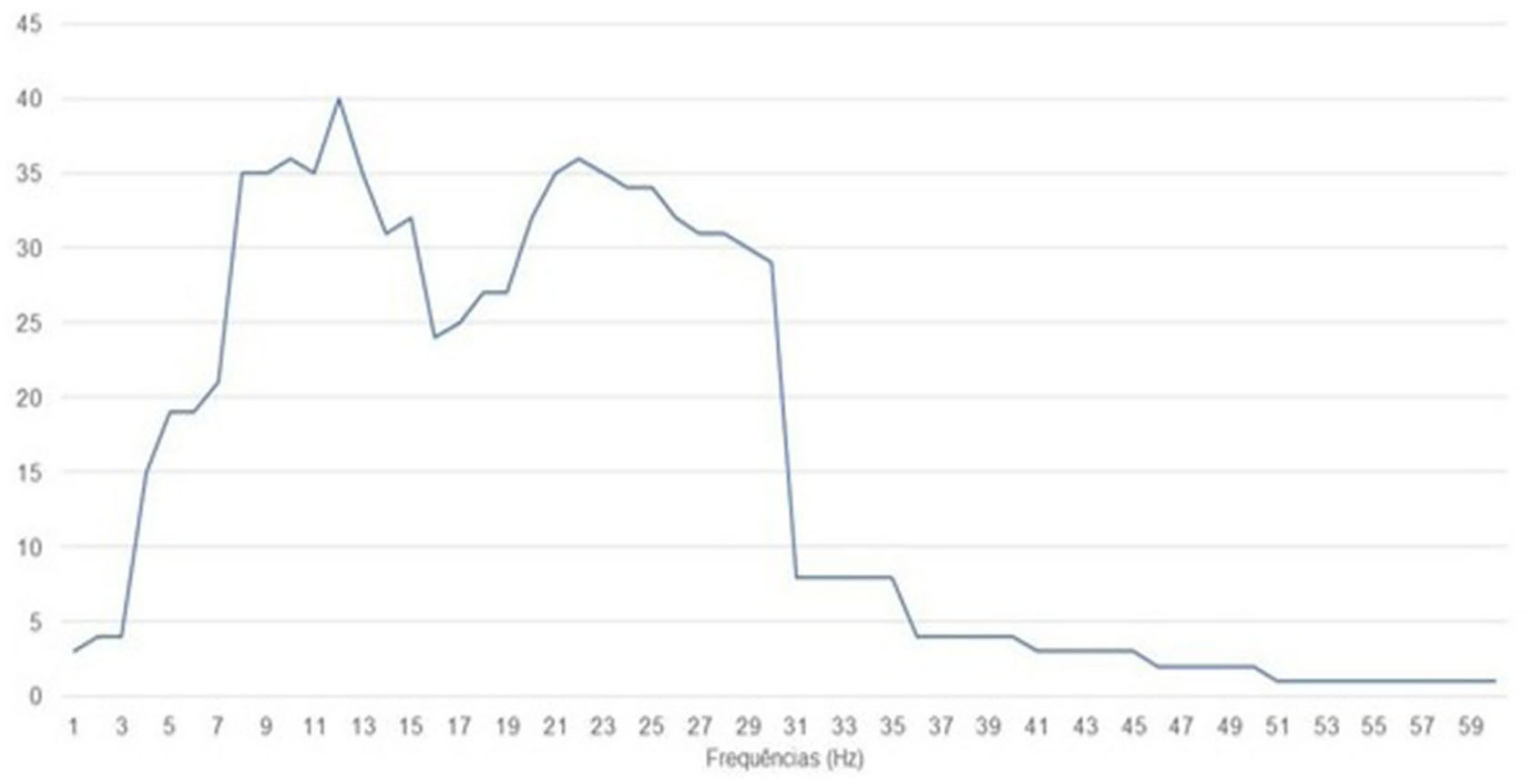
Frequencies used among the studies.

The studies focused on stroke presented motor imagination as the main task during training. The same was seen in the studies with Spinal Cord Injury, Amputation of Lower Limbs, Quadriplegia, and Paraplegia. On the frequencies used among the studies, it can be observed that the most used was 12 Hz, being used in 40 studies, being present in 23 stroke studies, followed by 10 and 22 Hz present in 36 articles. Figure 4 represents the sum of the frequencies used among the 44 studies.

Regarding the number of channels present in the studies, we observed the presence of studies using 60 channels (Tangwiriyasakul et al., 2014), and one channel (Kayiran et al., 2010; Cowley et al., 2016; Vučković et al., 2019), such results are present in different protocols within the set of the same disease. In terms of the neuroimaging technique used, the way in which the data is processed can be found described in Supplementary Table 2.

### 3.2 Different clinical applications

**Figure 5:**
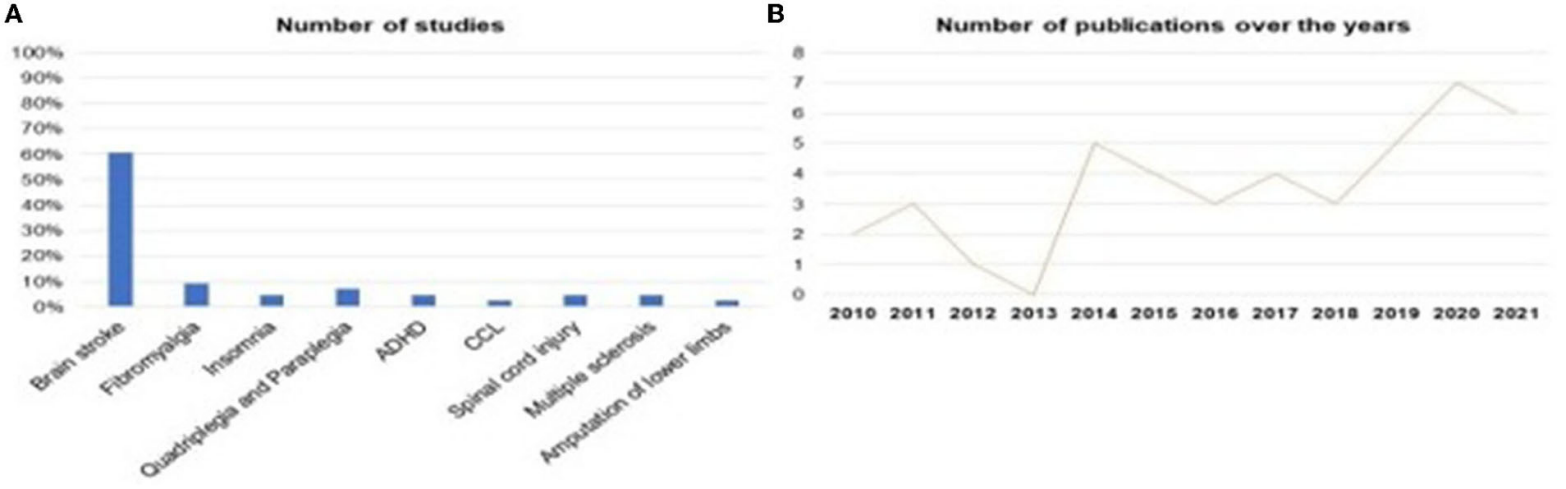
Representation of the studies among the illnesses and distribution of studies in years.

Was identified that SMR-based neurofeedback is being evaluated in different neurological/psychiatric pathologies. 60.5% of studies investigated SMR neurofeedback as a possible intervention in stroke, followed by 9.3% of studies in Fibromyalgia, 4.7% of studies in Insomnia, 4.7% of studies in Multiple Sclerosis, 2.3% of studies in Amputation of the Lower Limbs, 7.0% of the studies were about Quadriplegia and Paraplegia and 4.7% about Spinal Cord Injury, Figures 5A, B, represents the distribution of research published over the years, in which it is possible to see that the year 2013 had the lowest publication rate, while 2020 was the year with more research published, evidencing the growth of interest in the theme (Figure 5).

Among the results presented by the studies, there was an association between the beta and alpha bands and their relationship with the learning processes in terms of a closed circuit between the sensorimotor cortex and the paralyzed limbs (Carino-Escobar et al., 2019; Chen et al., 2020). The presence of assessment of cortical rhythms and slow cortical potentials was verified to investigate the onset of voluntary control of the lower limbs (Ibáñez et al., 2014).

Another aspect investigated refers to the presence of increased alpha and beta desynchronization in the ipsilesional hemisphere, as well as the potential to induce intrinsic ipsilesional SMR reorganization and mμ rhythm desynchronization in the ipsilesional hemisphere in impaired limb movement attempts (Pichiorri et al., 2015; Remsik et al., 2019; Tsuchimoto et al., 2019). The presence of immediate and long-term improvements in pain in protocols aimed at decreasing alpha bands, and increasing beta as well as ndalpha potency at C4 in patients with fibromyalgia (Kayiran et al., 2010). The impact of neurofeedback training on cortical areas called the pain matrix was verified using single-channel EEG (Hasan et al., 2021).

In studies with patients diagnosed with Multiple Sclerosis, the presence of improvement in cognitive functions was seen, such as in short- and long-term verbal memory, short-term visuospatial memory, working memory, and in the functional connectivity of patients with Multiple Sclerosis (Kober et al., 2019; Pinter et al., 2021). Hammer et al. (2011), showed improvements in the quality of sleep of patients with insomnia after neurofeedback training, but, such improvements were not visualized by Schabus et al. (2017), not being more efficacious than Cognitive Behavioral therapy.

Regarding the benefits provided by the feedback modalities, the presence of the use of an exoskeleton was verified as a way to assist in the execution of the movement and to provide feedback, in which the hybrid use of technology has been seen as a way of allowing an increase in the amplitude of movement. Task-related movement allows for long-term improvements, being used in cases of stroke and quadriplegia, and paraplegia (Onose et al., 2012; Grimm et al., 2016; Belardinelli et al., 2017; Chen et al., 2020).

Veikko et al. (2021) emphasized that the learning process itself can provide improvements in patients with ADHD, emphasizing the conceptualization model of learning as being the acquisition of a skill and not just operant conditioning. However, while there were improvements in symptoms for patients with ADHD obtained by self-report when compared to the waiting list, no learning improvements were observed on a computerized attention test (Cowley et al., 2016).

### 3.3 Experimental quality analysis

The mean is represented by the ratio between the sum of the data and the amount of data to be analyzed, thus, the mean of the items that performed the pre-experimental record is below 50% of the evaluated articles, representing a standard deviation of 24.05%. It is important to emphasize that the blinding of the studies is a way to guarantee that the participants, researchers and the study group responsible for investigating the outcomes cannot intervene in the results, thus reducing different risk of biases. Another important aspect is the lack of a control group as they are important to show the real effect compared to another intervention (Rosario Filho, 2020).

As for feedback specifications, an average of 88.63% (SD 15.33%) of the studies presented some type of feedback provided to the patient during training, an average of 83.33% (SD 19.65%) of the studies presented measures of neural signals r. An average of 87.5% (SD 23.29%) of the studies presented behavioral measures. It is also noted that, on average, only 4.54% of the studies provided data.

On average, only 4.54% of the studies provided online data from the EEG collection or the scales that were used before and after the interventions. The same problem was found with regard to the presentation of items that are between essential and non-essential, in which only about 11.03% of the analyzed works presented items considered non-essential for the presentation of results and consequently resulted in the quality of the works presented. The analysis demonstrated the presence of greater supply of information in items related to the type of feedback presented, the presence of collection of neural and behavioral signals. Figure 5 describes the results presented in each area.

**Figure 6:**
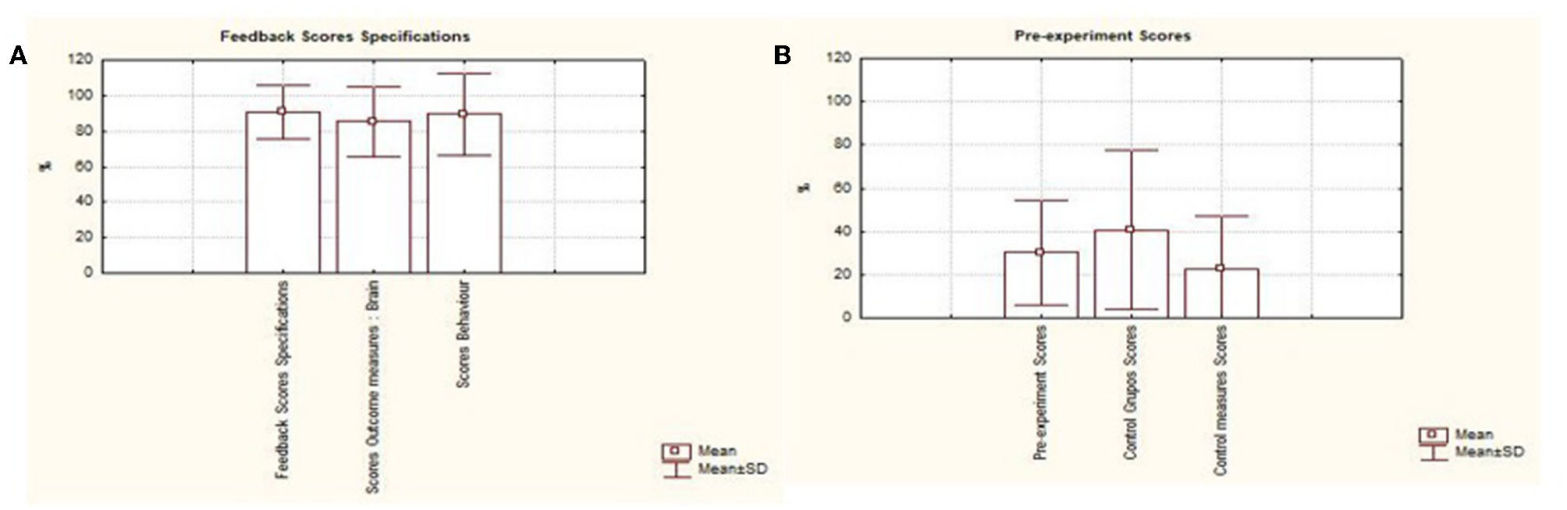
Describes the results presented in each area. Graphic presentation of the performance of studies based on the consensus on the reporting and experimental design of neurofeedback studies (CRED-nf), distributed among the items. **(A)** Feedback specifications; **(B)** pre-processing scores; **(C)** scores of essential and non-essential items.

The results were organized and separated according to the percentage data represented by each disease, this was based on the items considered by the CRED-nf as essential and non-essential and distributed in neurological diseases and neurodevelopmental disorders. Thus, with regard to neurological diseases, based on the figure below, the presence of studies that investigated the applicability of neurofeedback in cases of insomnia and fibromyalgia can be seen. Figures 6A, B indicate the percentage of essential and non-essential items present in each study, in which it is noted that insomnia publications met more essential criteria, an approximate average of 84% of the items, while works on fibromyalgia represented an average of 63% of the essential items.

It was possible to notice differences between the articles regarding the number of patients, the study by Wu et al. (2021) had a total of 60 participants, followed by Frolov et al. (2017), with a (*N*) sample of 55 participants, Marlats et al. (2020), with 33 participants and Schabus et al. (2017), with a total of 30 participants, the other works presented an experimental (*N*) of <30 participants. Thus, the size of the groups shows great variability in view of the number of participants present between the studies, mainly because they present the limited number of participants as a limiting factor for the studies.

The studies present variability based on the years of publication, that is, the studies with the largest number of participants dating from the years 2017, 2020, and 2021. The characteristics of the population, scales used between studies, protocols used to contain the number of sessions, and the channels used are present in our Supplementary material.

**Figure 7:**
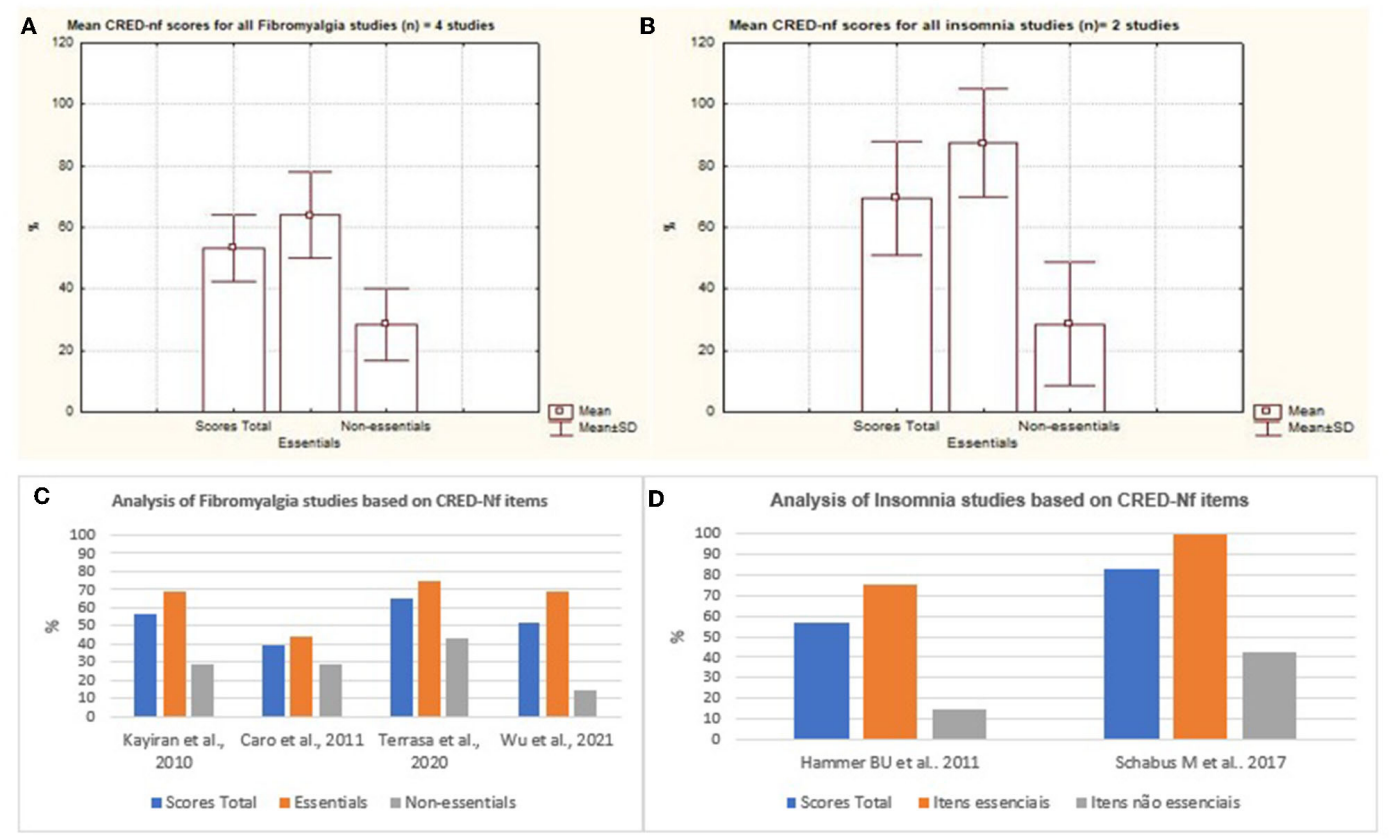
Representation of the mean and standard deviation of essential and non-essential items and total score between studies. Representation of the mean and standard deviation of essential and non-essential items and total scores of insomnia and fibromyalgia. **(C, D)** represent the distribution of essential and non-essential items and the total score between studies.

The results were organized and separated according to the percentage data represented by each disease, based on the items considered by the CRED-nf as essential and non-essential and distributed in neurological diseases and neurodevelopmental disorders. Thus, with regard to neurological diseases, based on the figure below, the presence of studies that investigated the applicability of neurofeedback in cases of insomnia and fibromyalgia can be seen. Figures 7A, B indicate the percentage of essential and non-essential items present in each study, in which it is noted that insomnia publications met more essential criteria, an approximate average of 84% of the items, while works on fibromyalgia represented an average of 63% of the essential items.

The study presented by Schabus et al. (2017) fulfilled 100% of the essential items and 40% of the non-essential items, representing better methodological rigor used in its approach. The authors discussed the effects of neurofeedback on memory and sleep, in a double-blind, placebo-controlled study in patients with insomnia (Schabus et al., 2017). The results showed that neurofeedback is not effective for the treatment of primary insomnia and therefore cannot be recommended as an alternative to Cognitive Behavioral Therapy for insomnia, which is currently considered the standard non-pharmacological model today (Schabus et al., 2017).

**Figure 8:**
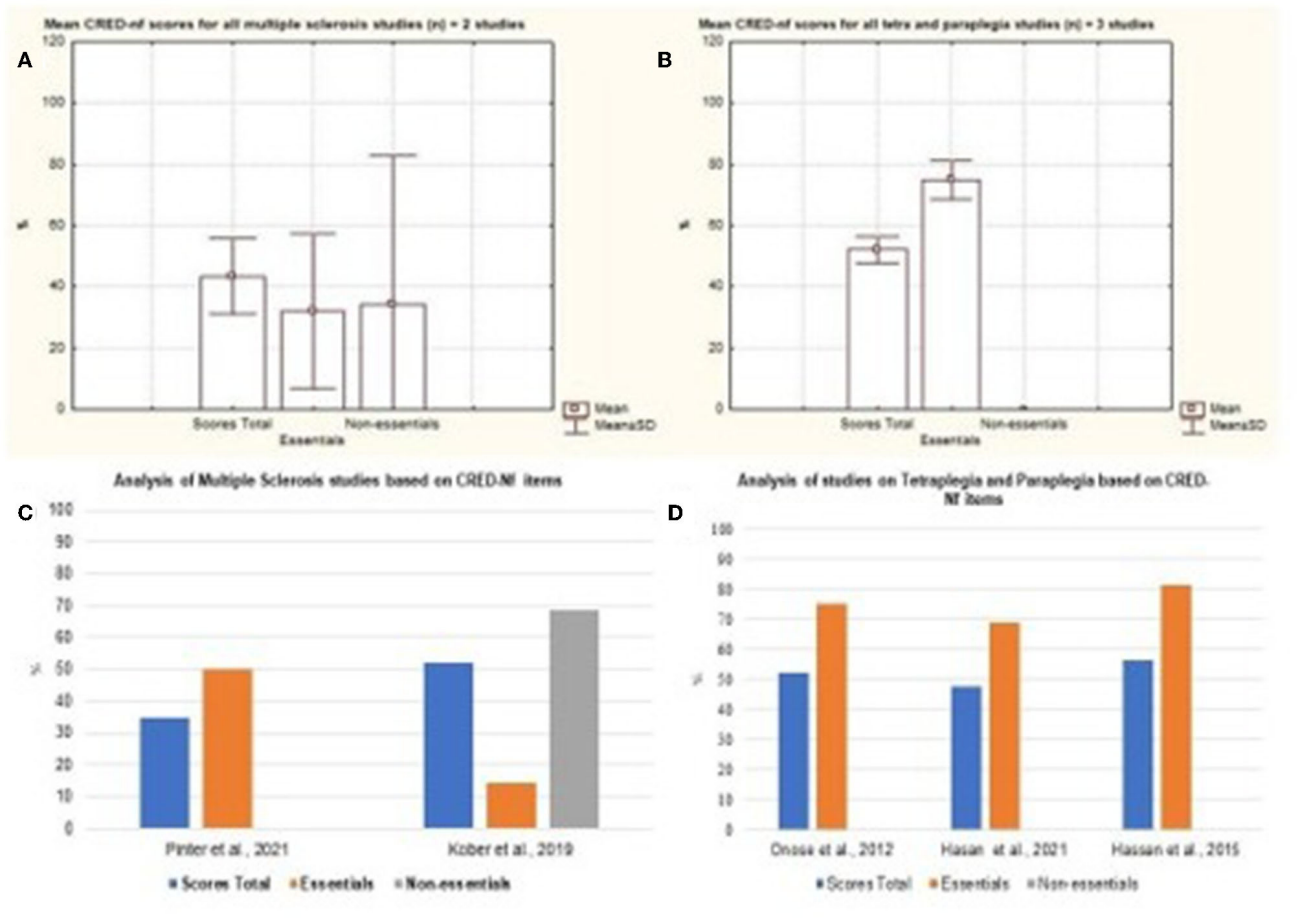
Representation of the mean and standard deviation of Multiple Sclerosis; Paraplegia and Quadriplegia; Tetraplegia and Paraplegia. **(A)** Representation of the mean and standard deviation of Multiple Sclerosis; **(B)** mean and standard deviation of Paraplegia and Quadriplegia; **(C)** representation of the essential and non-essential items of Multiple Sclerosis articles; **(D)** representation of the essential and non-essential items of articles on tetraplegia and paraplegia.

**Figure 9:**
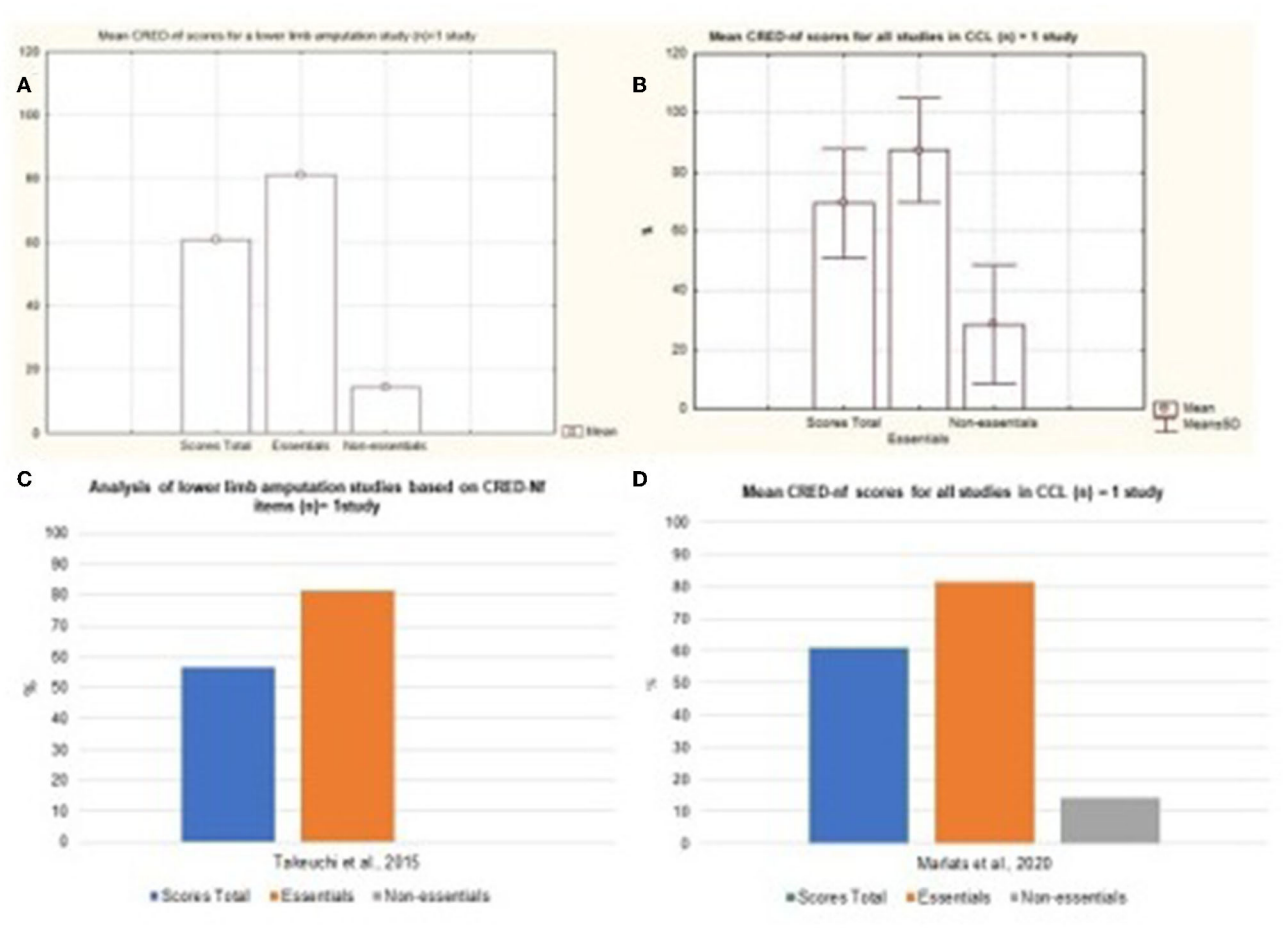
Representation of the mean and standard deviation of Lower Limb Amputation and Mild Cognitive Impairment study. **(A)** Representation of the mean and standard deviation of Lower Limb Amputation; **(B)** mean and standard deviation of the Mild Cognitive Impairment study; **(C)** representation of the essential and non-essential items of the Lower Limb Amputation article; **(D)** representation of the essential and non-essential items of the Mild Cognitive Impairment articles.

Diseases such as Spinal Cord Injury, Stroke, Quadriplegia, Paraplegia, and the Amputation of lower limbs represented most of the published studies. The Figure 8 represents the mean and standard deviation referring to the studies of Tetraplegia, Paraplegia, and Multiple Sclerosis. The figures represent the Mild Cognitive Impairment and Lower Limb Amputation charts (Figure 9). It is noted that only one study of both diseases was analyzed and that in both cases around 80% of the essential items were presented.

**Figure 10:**
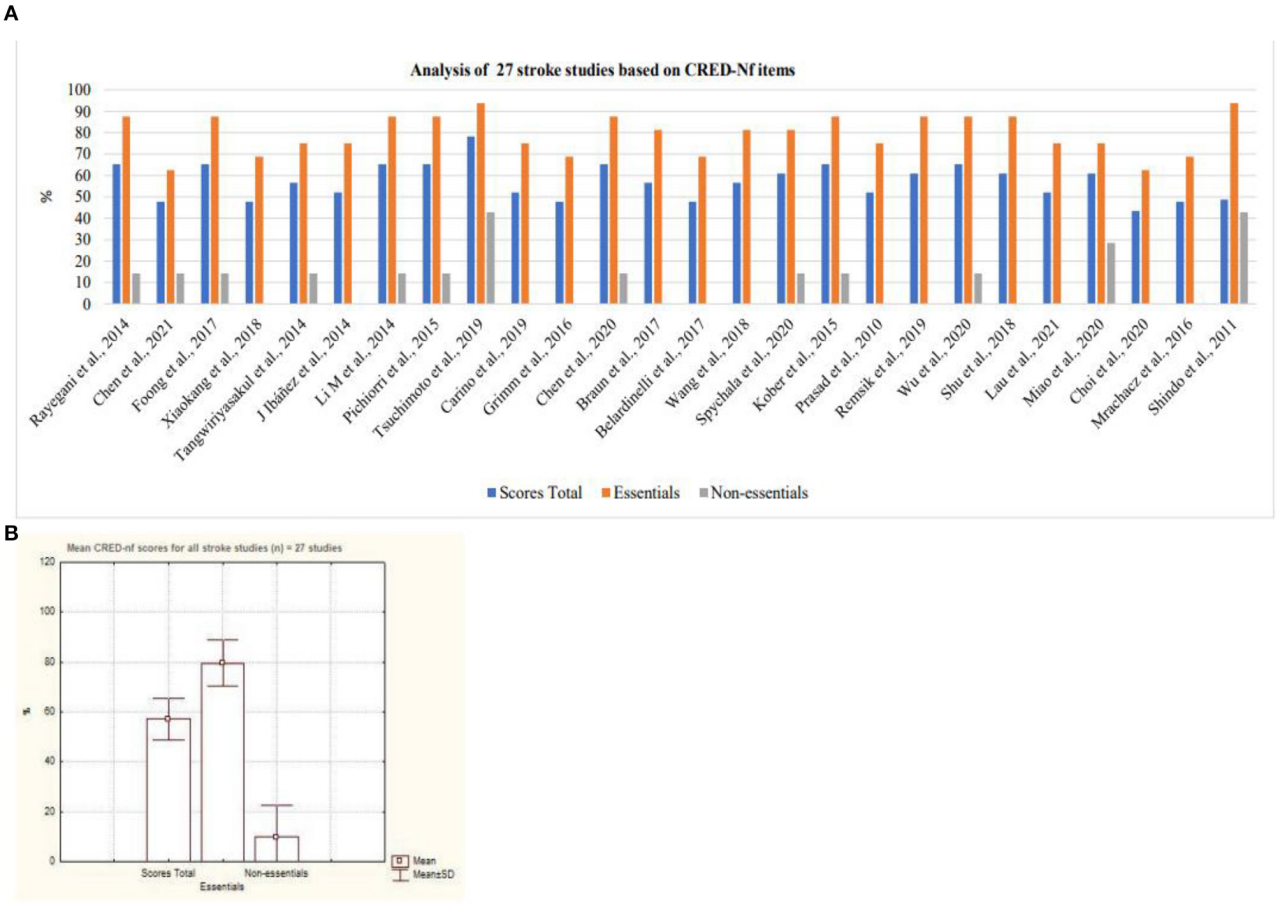
Analysis of stroke studies based on CRED-Nf items. **(A)** Representation of twenty-seven stroke studies containing essential and non-essential items; **(B)** mean and standard deviation of twenty-seven stroke studies.

**Figure 11:**
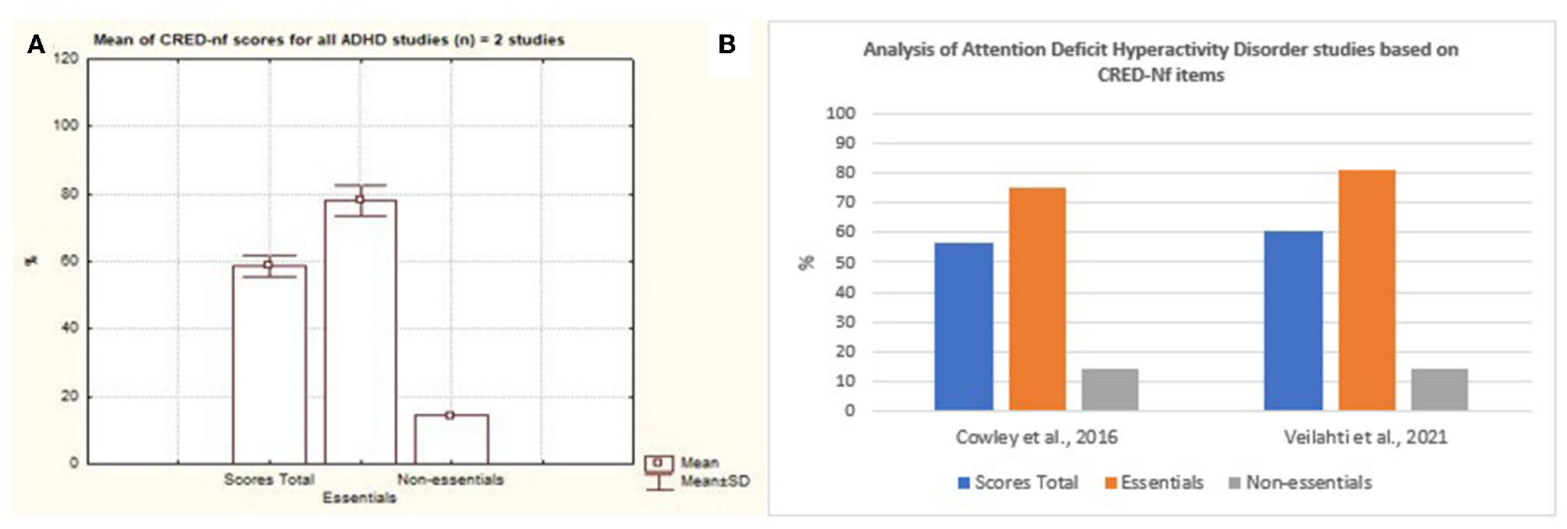
Representation of the studies of Attention Deficit Hyperactivity Disorder containing essential and non-essential items. **(A)** Mean and standard deviation of the two articles on Attention Deficit Hyperactivity Disorder. **(B)** representation of the two studies of Attention Deficit Hyperactivity Disorder containing essential and non-essential items.

Cerebral Vascular Accident was considered the disease with the largest number of scientific studies that address neurofeedback based on SMR signals as a form of intervention (Figure 10). Nevertheless, it is possible to observe a variation between the quality of the studies presented with regard to essential and non-essential items. As for neurodevelopmental disorders, only two studies that focused on ADHD were analyzed. Based on Figure 11, it is possible to observe that both studies contemplated more than 50% of the items considered by the CRED-nf as essential. Veikko et al. (2021), reported ~81% of the items.

Veikko et al. (2021) investigated, through a controlled and randomized clinical trial, the effects of the learning process on positive performance during neurofeedback training, in which they supported the concept of the importance of the learning process based on neurofeedback as a way of acquiring skills not just a result of the operant conditioning process.

### 3.4 Quantitative analysis of clinical improvement

**Figure 12:**
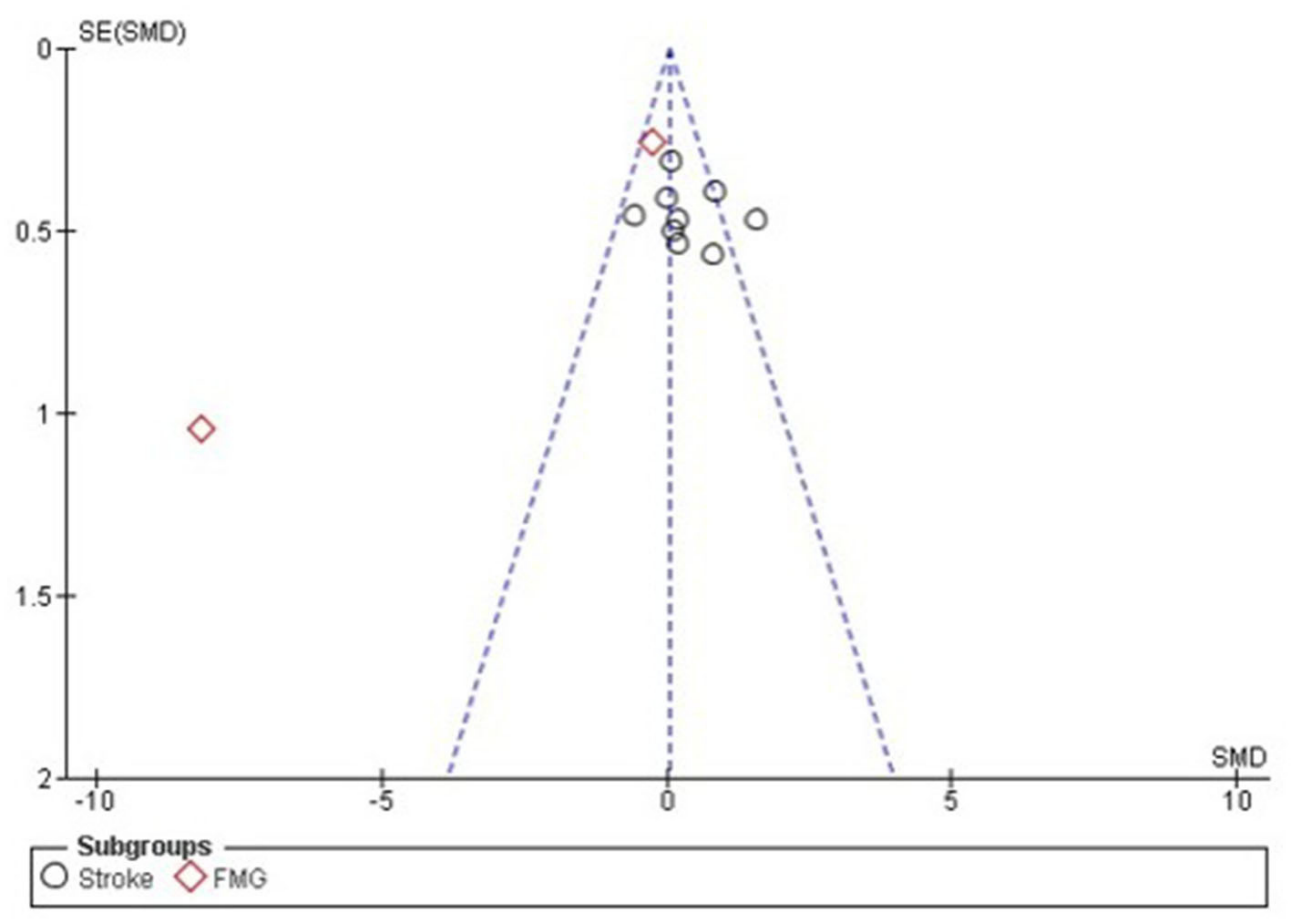
Forest plot: caption funnel plot of the study distribution. Stroke: study that used patients with cerebrovascular accident (CVA); FMG: studies with patients with fibromyalgia (FMG).

Statistical analysis did not show greater benefit in stroke patients when compared to other therapies such as medication and occupational therapy as the results show an increase benefit in the control arm compared to intervention (Std mean dif. 0.31, 95% CI 0.03–0.60, *p* = 0.03). It was possible to visualize the absence of significant heterogeneity between stroke studies, being classified as moderate *I*^2^ = 46% *p* = 0.06. The lack of standardization between the scales used, and the absence of a measurement scale after the interventions that compared the patient's performance before and after the interventions, hampered the statistical analysis (Figure 12).

**Figure 13:**
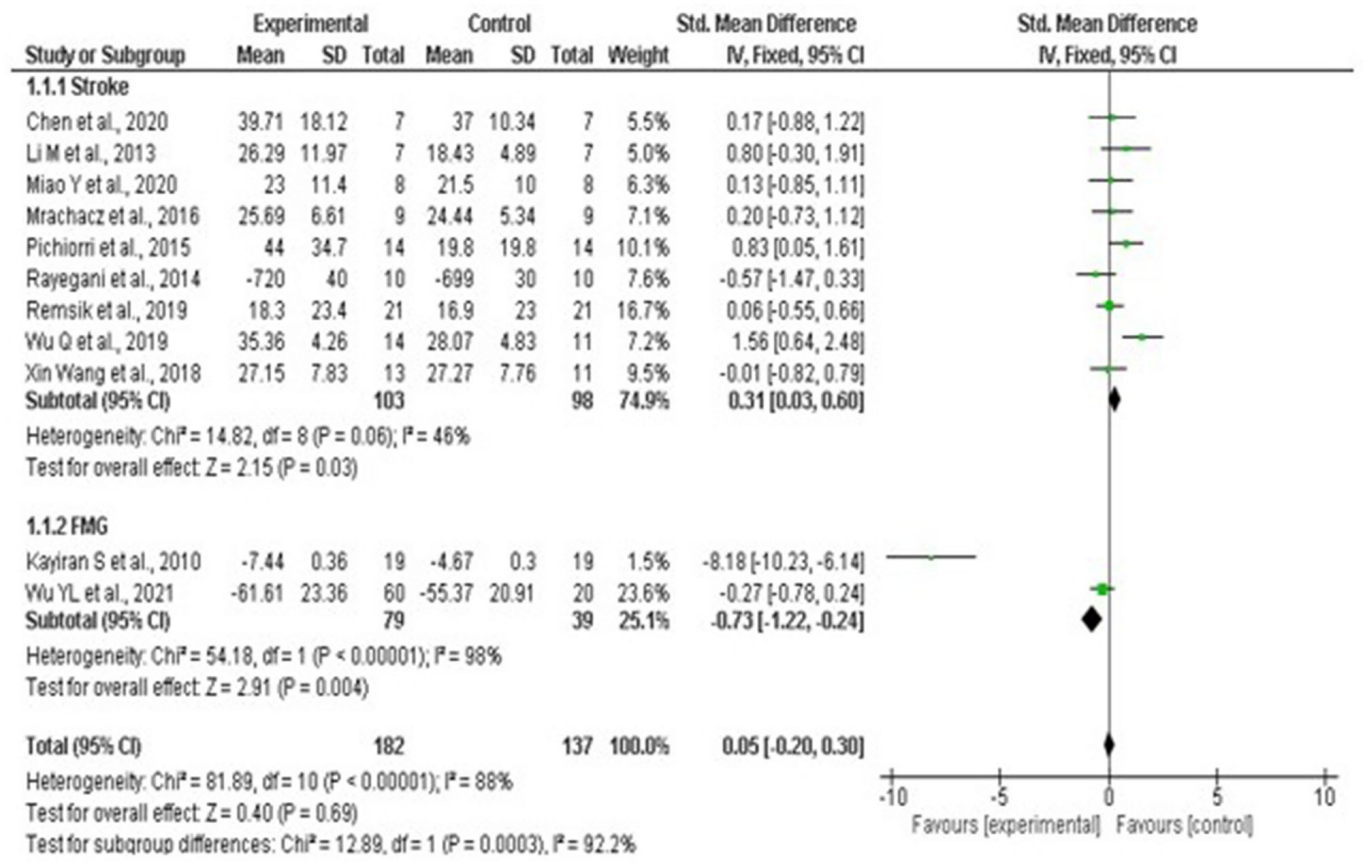
Forest plot separated by subgroups. Stroke studies, FMG studies and general.

With regard to studies with patients diagnosed with Fibromyalgia, it is noted through the quantitative analysis the presence of greater benefit for the group that used neurofeedback (Std mean dif. −0.73, CI 95% −1.22 to −0.24, *p* = 0.001). Nevertheless, when performing the overall assessment across studies, no significant differences were observed between the use of neurofeedback and standard therapy (Std mean dif. 0.05, 95% CI, −0.20 to 0.30, *p* = 0.69), with the presence of substantial heterogeneity *I*^2^ = 92.2%, *p* < 0.001. For the effect measurement analysis, the “standard mean difference” was used due to the presence of variability between the measurement scales used between the studies, as described in Supplementary Table 3. Figure 12 represents the Forest plot of the analyzed studies, and Figure 13 represents the studies separated by subgroups.

## 4 Discussion

In this first systematic review with meta-analysis of SMR-based EEG neurofeedback studies, we evaluated the quality of the studies and the effectiveness of this technique in different pathologies. Although authors such as Onose et al. (2012), Takeuchi et al. (2015), Schabus et al. (2017), Vučković et al. (2019), Terrasa et al. (2020), and Veikko et al. (2021) report the presence of clinical improvements during interventions, we did not find evidence to support this findings. This shows the requirement of randomized studies with more comprehensive experimental (*N*) and with greater methodological rigor.

Therefore, it was noted that current studies have investigated the mechanisms of use of neurofeedback from a multidisciplinary perspective through experimental and clinical paradigms, with the aim of understanding its scope from the point of view of neuroscience, neuroengineering, and learning sciences (Sitaram et al., 2017).

However, a lack of standardization was identified between the applied protocols, an element that hinders the analysis of the quality of the published studies, especially in regards to the experimental conditions, the benefits, and side effects, which end up generating a lack of adherence to the treatment. In relation to side effects, studies that reported such elements highlighted the presence of fatigue, abandonment without reporting the reason (Rayegani et al., 2014), and allergy to the gel (Li et al., 2014).

It is important to emphasize that the control conditions are essential, as they allow for greater reliability in the results, allowing the analysis of unspecified effects and, consequently, the comparison between other therapeutic approaches (Trambaiolli et al., 2021). The intervention group should also be monitored, as when followed it is possible to provide a more robust experimental design of the neurofeedback experimental reports being intended for all modalities (Ros et al., 2020).

Thus, to compare the clinical effect between groups in neurofeedback studies with different diseases, the studies were organized according to CRED-nf indications for quality analysis. Thus, the 44 surveys evaluated were based on the CRED-nf parameters separated into essential and non-essential items. The results showed an absence of standardization between experimental and control groups.

Trambaiolli et al. (2021), when demonstrating the importance of larger samples to visualize specific effects of neurofeedback, such reflections are also pointed out in the studies analyzed as a limitation present in the studies (Rayegani et al., 2014; Chen et al., 2020; Foong et al., 2020). In addition, the follow-up period of the studies was varied and not applied in all cases. The same applies to the number of sessions that do not have a specific standardization across articles as specified in Supplementary Table 3.

The investigation of the effects of neurofeedback between each session, and especially the use of psychosocial assessment scales to observe the patient's motivation during training, should be investigated, as these elements, as highlighted by Enriquez-Geppert et al. (2017) may influence the patient's performance and learning process. It should be noted that follow-ups after training are not standardized because, in addition to few studies following patients after training, the time varies between 7 days (Bismuth et al., 2020); 2–4 weeks (Schabus et al., 2017; Chen et al., 2020), 6 months (Wang et al., 2018), and 6-year follow-ups (Caro and Winter, 2011).

With regard to side effects or number of withdrawals, no side reactions were observed during training, on the other hand, it can be seen that many of the withdrawals were related to personal reasons that prevented them from completing, not following instructions, technical equipment failures (Kayiran et al., 2010; Hammer et al., 2011; Cowley et al., 2016; Tidoni et al., 2017; Foong et al., 2020; Terrasa et al., 2020; Veikko et al., 2021).

Moving on to the analysis of the quantitative aspects of the articles evaluated, it was possible to verify that of the 44 articles evaluated, only 11 could be used in the statistical analysis, due to the absence of pre- and post-intervention scales, small number of participants or articles between the pathologies and lack of standardization of the scales used. It was observed that the largest number of published studies are focused on stroke (60.5%) and fibromyalgia (9.3%), with analysis of clinical efficacy based only on these two pathologies.

Nonetheless, authors such as Krylova et al. (2021), question the importance of seeking larger samples, cost, and variability of groups, in addition to biases in sample size estimates, so that it is possible to visualize the minimum number of participants to determine the sample power and the difficulty of getting larger groups in practice, mainly due to the demand for several sessions. However, even if the power number cannot be achieved, it must be declared together with the power calculation of the analysis as recommended by the CRED-nf, in order to preserve the quality of the work developed (Ros et al., 2020).

Thus, this study showed the importance of developing more robust studies to investigate the clinical benefits of neurofeedback based on sensorimotor rhythm, with greater methodological rigor, and standardization of protocols, channels, and equipment used during training. Therefore, this study clearly showed the scientific area's interest in the benefits of neurofeedback based on sensorimotor rhythm for various pathologies, but that currently there is still no consensus on its benefits, mainly due to the low methodological rigor.

Statistical analysis showed that studies carried out with patients diagnosed with stroke did not demonstrate greater benefit than other therapies, as described by Wu et al. (2020), who demonstrated in their study with 25 patients with subacute stroke with moderate paralysis severe, that patients in both groups (intervention and control), showed significant improvements in both groups, not demonstrating superior performance for neurofeedback. Cao et al. (2022) verified the effects of space-time analysis and network analysis as a way to improve the performance of patients with stroke, in which positive effects were found when analyzing the performance obtained in a motor scale, in which six patients out of seven showed improvement.

Rimbert and Fleck (2023) identified that the repetitive and prolonged practice of training with BCI based on motor imagination did not decrease the patient's sense of wellbeing and was able to generate a sense of automation in performing tasks. The authors emphasize the importance of developing larger and long-term studies that seek to investigate how brain motor patterns change over time in the same individual and how intrapersonal factors can influence performance during training (Rimbert and Fleck, 2023).

The analysis of studies with patients diagnosed with Fibromyalgia showed greater benefits for the intervention group. There is a higher methodological quality of studies when they are analyzed based on the CRED-nf and compared with the results of publications on stroke. However, when the overall assessment of the studies was performed, no significant differences were found between the use of neurofeedback and standard therapy. Fibromyalgia studies have shown that neurofeedback can be beneficial for pain, fatigue, depression, and quality of life, but they highlight the small number of participants as a limiting element (Kayiran et al., 2010).

Thus, since the samples are small, the studies have low external validity and it is not possible to draw conclusions based on evidence about the usefulness of the method for these clinical indications so far. The main limitation of this research is related to the great variability between studies, due to the lack of standardization regarding the number of participants and degrees of disease severity, as well as the scales to be used pre- and post-intervention. Such elements hampered the development of the quantitative analysis, as, even though 44 studies were analyzed in the qualitative phase, only 11 provided the necessary information for the statistical analysis. The absence of analysis of the references of the studies included in the study.

## 5 Final considerations

In conclusion, although current studies that focus on the application of neurofeedback based on sensorimotor rhythm exist, such studies present numerous limitations mainly related to the sample (N), methodological rigor, and lack of standardization of outcomes. Consequently, it has become necessary to carry out research that seeks to overcome such obstacles in order for it to be possible to understand the application of neurofeedback based on the electroencephalogram, in the sensorimotor areas, to have the clinical potential to substitute the therapeutical standard we use today. The results demonstrate that neurofeedback can be beneficial to patients with fibromyalgia and must be further investigated with a larger group of patients to confirm these results.

## Data availability statement

The original contributions presented in the study are included in the article/Supplementary material, further inquiries can be directed to the corresponding author/s.

## Author contributions

TR and MC carried out the conception, design, and writing of this study. TR, MC, and EP searched the databases, selected articles, and extracted data, and performed data acquisition, analysis, and interpretation. TR and AG prepared the main manuscript. MC and HT critically reviewed the manuscript. All authors approved the final version of the manuscript.
