# Supplementary material for: Clinical applications of neurofeedback based on sensorimotor rhythm: a systematic review and meta-analysis
Source: Front Neurosci. 2023 Nov 20;17:1195066. doi: 10.3389/fnins.2023.1195066 (PMC10694284; doi:10.3389/fnins.2023.1195066)
Supplement: Supplementary file 1 [file Data_Sheet_1.PDF]

Supplementary Material

CLINICAL APPLICATIONS OF NEUROFEEDBACK BASED ON SENSORIMOTOR RHYTHM: A SYSTEMATIC REVIEW AND META-ANALYSIS.

Tatiana Ferri Ribeiro[1], Marcelo Alves Carriello[2*], Eugenio Pereira de Paula Júnior[1,2], Amanda Carvalho Garcia, Guilherme Luiz da Rocha, Helio Afonso Ghizoni Teive.

*** Correspondence:** Corresponding Tatiana Ferri Ribeiro - psitatianaferri@gmail.com

1 Supplementary Data

TABLE 2 - Population characteristics and values of scales used pre and post.

Study	**Population**	**Age (years baseline mean ± standard deviation)**	**Education (years)**	**Pre/Post Symptom Scales (baseline mean ± standard deviation)**	**Comorbidity and treatment**
Stroke					
Girijesh Prasad et al., *2010*	GE: n=5	GE: 58,6±8,98	NI	NA	NI
Shindo K et al., *2011*	GE: n=8	GE: 46±68 (intervalo)	NI	SIAS knee Mouth: 168 (média)<br>SIAS knee Mouth: 23 (8 pessoas)	Hypertension (N=8)
Li M et al., *2013*	GC: n=07<br>GE: n=08	GC: 60±76<br>GE: 65±74	NI	NA	NI
Rayegani et al., 2014	GE:. n=10<br>GC: n=30	GE. 51 anos.<br>GC: 54±53	OT group (5 uneducated, 2 basic, 1 undergraduate and 2 postgraduate). OTBF group (5 uneducated, 3 basic, 1 undergraduate, 1 postgraduate); OTBF group (5 uneducated, 2 basic, 1 EM, 1 graduate)	Grupo OT 175 (32); OTBF 165 (62); OTNF 120 (40); OTBF 125 (44)	NI
J Ibáñez et al., *2014*	GE: n= 08<br>GC: n=6	52 ± 83	NI	NA	NI
Tangwiriyasakul et al., *2014*	GE: n=10<br>GC: n=05	26,4	NI	Upper Extremity Fugl-Meyer Scale; Ranging: 0 to 66; Scores between: 4 / 65	NI
Pichiorri et al., *2015*	GE: n=14<br>GC: n=14	GE: 64,1<br>GC: 59	NI	GE: Experimental<br>Test: (0.76 mean);<br>Modified Rankin scale: pre 2.08;<br>Stroke scale: (pre 24, 92)<br>FMA: pre 2.00<br>GC: PR (1.16 mean);<br>(pre 1.75)	NI

				Stroke scale: (pre 26.44);<br>FMA: (pre 1.38);<br><br>GE: Experimental<br>Post Test: (0.84 average);<br>Modified Rankin scale: (post 2.00);<br>Stroke scale: (post 25.69);<br>FMA: (post 1.85);<br>GC: Post (1.09 mean);<br>(post 1.76);<br>Stroke scale: (post 24.44);<br>FMA: (post 1.39);	
Silvia Erika Kober et al., *2015*	GE: n= 11 SMR (12/15Hz); or Upper Alpha n = 6 (10–12 Hz), n = 7 stroke patients<br>GC: n= 40	GE: SMR 37± 74<br>GE: UPPER 53± 82<br>GE: Usual 49 ± 78<br>GC: 59.6	NI	NF (F (1.5) = 6.37, p = 0.05); SMR differed significantly from zero (t (10) = 2.38, p < 0.05). For the Upper Alpha group of patients, the regression model was significant (F (1.5) = 8.25, p < 0.05) and explained 67% of the variance in Upper Alpha power across the training runs .	NI
Florian Grimm et al., 2016	GE: n= 7	59 ± 9,3 [41 89] anos	NI	Fugl-Meyer (mUE-FMA) < 25	NI
Mrachacz* et al., *2016	GC: n= 13<br>GE: n= 09	GC: 23± 51<br><br>GE: 36± 68	NI	Control group: Mean mRS Scale (pre/post): 2.08 / 20.00<br><br>Fugl-Meyer scale (pre/post): 24.92/25.69;<br><br>ASS (Ashworth) scale (pre/post): 2.00 / 1.85<br><br>Experimental group: Mean of the mRS Scale (pre/post): 1.75/1.76<br><br>Fugl-Meyer scale (pre/post): 26.44/24.44<br><br>ASS scale (Ashworth) (pre/post): 1.38 / 1.39	NI
Niclas Braun et al., *2017*	GC: n= 20<br>GE: n= 20	GC: 60, 1(7,67)<br>GE: 59,1 (9,94).	NI	GEl: MOCA: 22,5 (5,63)	NI
Paolo Belardinelli et al., *2017*	GE: n= 9	57± 70	NI	FMA (pré/post): 16,23 ± 6,79 para 19,52 ± 7,91 (p = 0,0015)	NI
Foong et al., *2017*	GC: n=19<br>GE: n= 55	GC: 58.0 ± 52.0;67.0	NI	GC: ARAT (pré/post): 3.0 [0.0; 30.0] / 6.0 [0.0; 31.0].	NI

		GE: 58.0 ± 48.0;13.0		FMMA (pré/post): 12.0 [11.0;49.0] / 17.0 [12.0; 51.0]<br><br>GE: ARAT: 4.0 [0.0; 31.0] / 6.0 [1.0;43.0];<br><br>FMMA (pré/post): 24.0 [12.0; 40.0] / 29.0 [14.0; 47.0]	
Xiaokang Shu et al., *2018*	GC: n=10<br>GE: n=24	GC: 28 ± 5<br>GE: 48 ±14	NI	MMSE (pré / post]: 0-30 / 27-30.<br><br>FMA-EU (pré / post): 0-66 /4 – 54	NI
Xin Wang et al., *2018*	GC: Robot non−EEG_Text , n = 11 (treinamento da mão sem orientação)<br><br>GE: EEG_AO, n = n=13	GC: 30 ± 69<br><br>GE: 40 ± 59	NI	GC: FMA – EU: (pre / post): 16 [41] / 14 [41]; after 6 months 18 [32].<br><br>GE: FMA – EU: (pre / post): 13 [34] / 17 [39]; after 6 months 16 [50].	NI
Xiaokang Shu et al., *2018*	GE: n=11	GE: 25 ± 66	NI	MMSE: 28 ± 30<br><br>FMA-EU: 4 ± 40	NI
Tsuchimoto et al., *2019*	GC: n=18<br>GE: n=18	GE: 58 ± 10	NI	FMA -EU: 28 ± 21<br><br>MMSE: 29 ± 2	NI
Carino-Escobar et al., *2019*	GE: n= 9	GE: 59,9 ±2,8	NI	FMA : EU (pre-post): 9± 59 / 12± 61.	NI
Alexander B. Remsik et al., *2019*	GE: n= 21	GE: 61,6 ± 15,3	NI	ARAT (Baseline): 26.6 ± 26.1<br>ARAT follow-up: 0-57<br>MMSE: 27.2(29) ± 3.8<br>MMSE follow-up: 28.3(29) ± 2.7	NI
Shugeng Chen et al., 2020	GC: n= 7<br>GE: n= 7	GC: 52.0 ± 11.1<br><br>GE: 41.6 ± 12.0	Medication: following the guidance of the rehabilitation doctor; (2) routine rehabilitation	Group control:<br>FMA – EU (pre/post): 32.3 (11.8) / 28 - 58<br>Fugl-meyer (post):<br>7.14%, p = 0.048	NI

			therapy: physical therapy (20 min, five times a week), low-frequency electrical stimulation (20 min, five times a week), occupational therapy (20 min, five times a week)	GE: FMA – EU (pre / post): 31.3 (11.5) / 14 - 65<br>Fugl-meyer (post): (12.77%, p = 0.032)	
Choi I et al., *2020*	GE: n= 8	GE: 46,0 ± 13,5	NI	NI	NI
Nadine Spychala et al., *2020*	GC: n=9<br>GE: n=9	GC: 60.22 ± 9.77<br>GE: 60.33 ± 9.31	NI	GE: MoCA: 21.77	NI
Miao Y et al., *2020*	GC: n= 8<br>GEl:n= 8	GC: 25- ± 72<br>GE: 22 ± 70	NI	GCe: FMA -EU: (pre / post): 20:6±9:7 21:5 ± 10:0<br>GE: FMA – EU: (pre/post)<br>19:5 ± 9:9 23:0 ± 11:4	NI
Wu Q et al., *2020*	GC: n=11<br>GE: n=14	GC: 64.82 ± 7.22<br>GE: 62.93 ± 10.56	Standard medical treatment and rehabilitation for 4 weeks, routine physiotherapy and occupational therapy focused on rehabilitation of arm and hand movements used in daily activities such as picking up a tube of toothpaste, eating, reaching and grasping in sitting and standing.	GC (pre / post): MMSE: 25.18 ± 2.86<br>FMA – UL: 14.09 +- 2.51 / 28.071 +- 4.832 / 8.36 +- 2.116<br><br>ARAT: 1.00 (0.00, 10.00) / 4.00 (3.00, 24.00) / 4.00(0.00, 4.00).<br><br>WMFT: 25.09 +- 2.96 / 28.00 (18.00, 50.00) / 3.00 (1.00, 14.00).<br><br>Experimental group (pre/ intra/ post):<br>MMSE: 24.29 +- 2.70<br><br>FMA – UL: 18.43+-2,645 / 35,357+-4,255 / 16.93 +- 2,560<br><br>ARAT: 9.50 (3.00, 23.25) / 28.07 +- 4.83 / 8.50 (4.75, 24.00)<br><br>WMFT: 30.07 +- 3.38 / 47.79 +-5.00 / 17.71 +-3.34	NI

Lau CCY et al., *2021*	GE: n=21	GE: 54 ± 8	NI	ARAT (pre/post/post 6 months)<br>11.3 / 17.3 / 15.7<br>FMA (pre / post / post 6 months):<br>21.0 /25.1 /25.0	NI
Chen et al., *2022*	GC: n=18<br>Grupo MI Task: n= 21	GC: 49.9±13.6<br>GE: 52.4±11.9	NI	MA Task Group: FMA – EU: 3.5±4.9<br>MI Task Group: FMA – EU: 3.4±6.9	NI
Insomnia					
Hammer BU et al., *2011*	GE: n= 8	GEl: 49.63	NI	ISI scale (pre/post): 17.13 (15,794,18,466) 6.56 (5,901, 7,220)<br>PSQI-T (pre/post): 13.38 (12,506, 14,254) 4.50 (4,194, 4,806)<br>PSQI-SE (pre/post): 77.64 (74.85, 80.43) 93.18 (91.87,94.49)<br>QOLI (pre/post): 46.13 (42,908, 49,352) 52.63 (49,827, 55,433)	NI
Schabus M et al., *2017*	EG: patients with insomnia: n=16<br>Misperception insomnia group: n=9<br>Sleep control subjects: n=26<br>GC: n=12	EG primary insomnia: 38.59.<br>NFB Group: 26.67<br>GC: 35.52	NI	Patients with insomnia: PSQI (pre/post)<br>TST: 358.0 / 371.0<br>SUN: 27.0/19.8<br>Patients with misperception: PSQI (pre/post)<br>TST: 382.5 / 390.0<br>SUN: 37.8 / 31.2	NI
Fibromyalgia					
Kayiran S et al., *2010*	GC: n=20<br>GE: n=20	GC: 31,78 ± 6,17<br>GE: 32,39 ± 6,72	NI	GC (NF / GC):<br>HAD scale 20.83<br>BDS scale: 26.00 / p<0.001<br>Scale: HAS: 19.72 / p< 0.001<br>BAS scale: 26.17 / p< 0.001<br>VAS pain scale: 9.11 / p<0.001	fatigue and pain<br>Escitalopram

				VAS fatigue scale: 9.19 / p< 0.001<br>GE: (baseline / friedman): Scale HAD 16.94 / p< 0.001<br>BDS scale: 21.50 / p< 0.001<br>HAS scale: 25.06 / p < 0.001<br>BAS scale: 35.56 / p < 0.001<br>VAS pain scale: 8.94 / p< 0.001<br>VAS fatigue scale: 9.00 / p< 0.001	
Caro XJ et al., *2011*	GC: n=63<br>GE: n=15	GC: 50.5 ± 13.9<br>GE: 66.7 ± 12.3	NI	GC (Pre / Post): CPT – Visual: -0.16 / +0. 08<br>EG: Subtest (Pre/Post) CPT – Visual: -0.64 / -0.69*; ADHD (pre/post): 0.18 ± 3.24 1.85 ± 3.71	Standard treatment failure<br>(with at least one non-steroidal medication and one low-dose tricyclic medication).
Terrasa JL et al., *2020*	GC: 8<br>Good-SMR group respond: n=4<br><br>Bad Group – SMR respond: n=5	G(SHAM): 56.25± 11.99<br>GGood- SMR answer: 53± 9.77<br><br>GBad – SMR answer: 54.75 ± 8.46	NI	Group (SHAM): TSK: 3813+-15.72<br>PVAQ: 48.13+-12.52<br>FIQ:40.81+-5.13<br>Numerical pain scale (pre/post): 0-100 / 0 - 100<br>Good-SMR Group Reply:<br>TSK: 51.8+-13.72<br>PVAQ: 51+-11.29<br>FIQ: 68.37+-20.2<br>Numerical pain scale (pre/post): 20-70 / 10-50<br>Bad Group – SMR reply:<br>TSK: 27.75+-12.15<br>PVAQ: 45.5+-8.89<br>FIQ: 65.16+-15.16<br>Numerical pain scale (pre/post): 60-70 / 50-100	Depression (GC: N=6)<br><br>Good-SMR Group reply: 04<br><br>Bad Group – SMR reply: 03<br><br>Analgesic/myorelaxant (88.24%), antidepressant (76.47%) and anxiolytic (70.59%).
Wu YL et al., *2021*	GC n=20<br>GE: n=60	GC: 42.2<br>GE.6	GC:<br>≤ High school: 06<br>Faculty: 11<br>≥ Graduate: 03<br><br>GE:<br>≤ High school: 16<br>Faculty: 39	GC: BAI (pre): 20.9; BDI (pre) 19.8<br>GE: BAI (pre): 22.5; BDI (pre): 21.1; Brief Pain Inventory (B = -1.35, SE = 0.46, p = 0.003) and pain interference (B = -1.75, SE = 0.41 , p < 0.001), total scores from the Pain Questionnaire	GC: CHD: 04;<br>Insomnia: 0;<br>Depression: 04 ;<br>Anxiety: 02; Panic:0;<br>Dry eyes: 0; Migraine: 1; Rheumatic disease: 1; Analgesics: 06;<br>Topamax: 09;

			≥ Graduate: 05	Impact of Fibromyalgia Reviewed (B = -16.41, SE = 3.76, p < 0.001), sleep onset latency (B = -25.33, SE = 9.02, p = 0.005) and Psychomotor Error in vigilance test (B = -1.38, SE = 0.55, p = 0.013).	Pregabalin: 07; Clonazepam: 01; Antidepressant: 03; Complementary hypnotic therapy: 07; Acupuncture: 0 Rehabilitation: 0; Traditional Chinese Medicine: 04 EG: CHD: 06; Insomnia: 03; Depression: 21; Anxiety: 07; Panic: 02; Dry eyes: 03; Migraine: 01; Rheumatic disease: 04; Analgesics: 14; Topax: 32; Pregabalin:27; Clonazepam:05; Antidepressant: 15; Complementary hypnotic therapy: 16; Acupuncture: 10; Rehabilitation: 09; Traditional Chinese Medicine: 13
spinal cord injury					
Müller-Putz GR et al., *2014*	GC: n= 10<br>GE: n=16	GC: 28,1<br>GE: 40,43	NI	NI	NI
Vučković A et al., *2019*	GE: n= 20	GE: 50,6 ± 14,1	NI	Visual Numerical Scale (VNS): 1-30	Pregabalin: n= 05<br>Gabapentin: n= 03<br>Tramadol: n=02<br>Duloxetine: n= 01<br>Nabline:-
CCL					

Marlats F et al., *2020*	GE: n=22	GE: 76.1 ± 5.9	GE: 14.9 ± 2.6	MMSE: 25.4 ± 2.8.<br><br>MOCA: 23.1± 2.5<br><br>MoCa, F = 4,78;<br><br>RAVLT, F = 3,675<br><br>Forward digit range F = 13.82;<br><br>Goldberg Anxiety Scale F = 4.54;<br><br>Wechsler Adult Intelligence Scale-Fourth Edition (WAIS-IV; F = 24.75,<br><br>Mac Nair score (F = 4.47; p = 0.016). EEG theta power (F = 4.44; p = 0.016) and alpha power (F = 3.84; p = 0.027)	Initial assessment with occupational therapy
Amputation					
Takeuchi N et al., *2015*	GC: n=6<br>GE: n= 6	GC: 51.0 ± 6.4<br>GE: 58,5 ± 10.9	NI	GE: Visual Analog Scale: 0-21	NI
Quadriplegia and Paraplegia					
Onose G et al., *2012*	GE: n=09	GE: 23 ± 51	NI	AIS Frankel<br>(in numbers<br>converted)<br>grade: 1 -3<br><br>sensory AIS<br>punctuation<br>(y/224): 52-162<br><br>AIS engine<br>punctuation<br>(s/100): 09- 38	NI
Hassan MA et al., *2015*	GE: n=07	GE: 50 ± 4,6	NI	EG: VNS scale (pre / post): 6-9 / 2- 6	Patients using medication were instructed not to switch during therapy.

Hasan MA et al., *2021*	GE: n=05	GE: 51 ± 3	NI	EG: VAS scale on the training day (BNF/DNF: P1 = 6/5, P2 = 6/5, P3 = 5/3, P4 = 7/4, P5 = 9/6)	There was no change in medications during the study.
Multiple sclerosis					
Kober SE et al., *2019*	GC: n=07<br>GE: n=07	GC: 41.0 ± 1.6<br>GE: 36.9 ± 4.2	GC: 14.4 ± 1.4<br>GE: 15.4 ± 1.3	Cognitive assessment: Control group (Pre / Post):<br>Verbal immediate feedback short-term memory (SRT): 48.10 ± 2.26 / 46.79 ± 2.27<br>Immediate retrieval short-term special visual memory (SPART): 56.93 ± 3.42 / 54.67 ± 3.93<br>Information processing speed (SDMT): 38.55 ± 6.20 / 38.53 ± 4.61<br>Information processing speed (PASAT): 46.69 ± 3.33 / 45.73 ± 3.78<br>Verbal delayed feedback long-term memory (SRT): 49.61 ± 3.08 / 50.53 ± 3.33<br>Delayed retrieval long-term spatial visual memory (SPART): 56.96 ± 3.84 / 54.07 ± 4.54<br>Executive functions (WLG): 46.30 ± 4.89 47.27 ± 3.93<br>Cognitive constructs: 51.41 ± 1.80 / 49.24 ± 3.24<br>Short-term memory: 51.41 ± 1.80 / 49.24 ± 3.24<br>Long-term memory: 54.39 ± 3.90 53.09 ± 4.10<br>Information processing speed and concentration: 41.17 ± 4.77 / 40.57 ± 4.24<br>Executive functions: 46.30 ± 4.89 / 47.27 ± 3.93<br><br>Cognitive assessment: Experimental group (Pre / Post):<br><br>Verbal immediate feedback short-term memory (SRT): 50.72 ± 2.73 / 53.99 ± 3.09<br>Immediate retrieval special short-term visual memory (SPART): 52.53 ± 3.03 / 58.35 ± 3.80<br>Information processing speed (SDMT): 39.23 ± 5.36 / 43.56 ± 4.97	NI

				Information processing speed (PASAT): 43.79 ± 7.08 / 47.79 ± 5.29<br>Delayed feedback verbal long-term memory (SRT): 43.31 ± 4.37 / 56.06 ± 2.05*<br>Delayed retrieval long-term spatial visual memory (SPART): 47.57 ± 4.31 / 56.86 ± 3.85*<br>Executive functions (WLG): 46.53 ± 3.71 / 53.43 ± 4.84*<br>Cognitive constructs: 51.73 ± 2.73 / 57.26 ± 3.48<br>Short-term memory: 51.73 ± 2.73 / 57.26 ± 3.48<br>Long-term memory: 43.96 ± 3.67 / 58.63 ± 3.45<br>Information processing speed and concentration: 39.81 ± 7.13 / 44.81 ± 5.86<br>Executive functions: 46.53 ± 3.71 53.43 ± 4.84*	
Pinter D et al., *2021*	GC: n=7<br>GE: n=1	GC: 41,0 ± 1,6<br>GE: 36,9 ± 4,2	GC: 14,4 ± 1,4 GE: 15,4 ± 1,3	GC: Cognition (BRB-N) (Pre/Post): 48.1 (3.6) / − 1.5 (3.5)<br><br>GE (Pre / Post): 44.4 (4.2) / 10.8 (3.0)	NI
ADHD					
Cowley B et al., *2016*	GC: n=29<br>GE: n=25	GC: 36.45<br>GE: 35.72	GC: Primary: 05<br>Secondary: 18<br>Tertiary: 06<br>GE: Primary: 06<br>Secondary: 15<br>Tertiary: 04	GC: Brown-ADHD Scale: 23 ADD: 06<br>GE: Brown-ADHD Scale: 21 ADD: 04	NI
Veilahti AVP et al., *2021*	GC: n=12<br>GE: n=11	GC: 30 ± 37<br>GEl: 26 ± 57	NI	GC: BIS scale: 9-19<br>DES Scale: 09-64<br>GE:<br>BIS scale: 7-19<br>DES Scale: 04-118	NI

Caption: The abbreviations GC* control group and EG** experimental group. NI*** not informed.

TABLE 03 - Hardware detail and data processing.

Hardware / Preprocessing

Author	*neuroimaging technique*	*Hardware/software de imagem*	*Position of channels*	*Channels (N)*	*Data pre-processing*	*processing software*
Rayegani et al., 2014	EEG	ProComp Infiniti – Biofeedback Neurofeedback System v6.0 (Thought Technology Ltd Co, Montreal, Quebec, Canada)	C3 as designed per international electrode 10-20 .	8	2 e 100 Hz SMR (12-18 Hz), theta (4-8 Hz) e beta (13-30 Hz)	IBM SPSS-18 (IBM, Armonk, NY).
Chen et al., 2020	EEG	The Omega force feedback device w.	Online	31	Filtered by a bandpass filter between 1 and 100 Hz.	SPSS version 23.0 (IBM Inc., Chicago, IL, United States).
Foong et al., 2019	EEG -EEG e nBetter	Neurostyle Brain Exercise Therapy Towards Enhanced Recovery (nBETTER).	In the international positioning of the 10-20 system: F3, F4, FC3, FC4, C3, C4, CP3, CP4, P3, P4, FT7, FT8, T3, T4, TP7, TP8, Fz, Oz, FCz, Cz, CPz , Pz, A1 and A2.	24	Filter Bank Common Spatial Pattern Algorithm (SFCF), described in details in [46]. In short, this band passes the EEG signal to various frequency bands within 4 to 40 Hz.	MATLAB®

Xiaokang Shu et al., 2018	EEG	BrainAmp Amplifier (Brain Products, Gilching, Germany.	extended 10-20 system	32	The low pass filter setting was 0 to 100 Hz with a sampling rate of 200 Hz, while a 50 Hz notch filter was used to decrease power line interference.	EEGLAB
Tangwiriyasakul et al., 2014	EEG	(TMS International, Holanda) foram feitas usando eletrodos Ag/AgCl.	international system 5-10<br>All electrodes in the first row (T7, FT7, F7, AF7, FP1, FPz, FP2, AF8, F8, FT8 and T8) and some electrodes in the second row (F5, AF3, AF4 and F6).	60	Between 0.5 and 30 Hz using a fourth-order Butterworth filter.	NI
J Ibáñez et al., 2014	EEG	Acticap, Brain Products GmbH, Germany.	31 positions (AFz, F3, F1, Fz, F2, F4, FC3, FC1, FCz, FC2, FC4, C5, C3, C1, Cz, C2, C4, C6, CP3, CP1, CPz, CP2, CP4, P3, P1, Pz, P2, P4, PO3, PO4, and Oz, all according to the International 10–20.	31	Linear phase filter (FIR filter, 15th order, 0.05 Hz < f1, 1 Hz > f2).	Matlab.
Li M et al., 2014	EEG	G.tec biosignal amplifier (G.tecGuger Technologies, Graz, Austria).	The channels were grouped into left SMC (FC3, C1, C3, C5, CP3), left parietal lobe (P3), right SMC (FC4, C2, C4, C6, CP4) and right parietal lobe.	16	Filtered in a certain band 8–30 Hz	NI

Pichiorri et al., 2015	EEG	BrainAmp; Brain Products, Gilching, Germany. Connection between the BCI2000 and the "virtual hands.	10-20 system	61	Bandpass filtered between 0.1 and 70 Hz	NI
Tsuchimoto et al., 2019	EEG e fMRI	EEG - MOBIlab; Guger Technologies, Graz, Áustria) fMRI - Scanner 3 T MR (Discovery MR750w, GE Medical Systems, Reino Unido).	The electrodes were placed 20 mm lateral and medial to the C3 (left) and C4 (right) sites closest to the hand region in the sensorimotor area according to the international 10-20 system.	5	Bandpass filter (0.5–100 Hz).	MATLAB (MathWorks, United States)
Carino-Escobar et al., 2019	EEG	g.USBamp biosignal amplifier.	Placed in positions F3, F4, Fz, P3, P4, Pz, C3, C4, Cz, T3 and T4 of the international system 10-20.	11	Bandpass filters in the following frequency bands: 8-12 Hz, 12-16 Hz, 16-20 Hz, 20-24 Hz, 24-28 Hz and 28-32 Hz, all are FIR type and order 30.	Matlab.

Florian Grimm et al., 2016	EEG	BrainAmp DC.	System 10–20 (FP1, FP2, F3, Fz, F4, FC5, FC3, FC1, FCz, FC2, FC4, FC6, C5, C3, C1, Cz, C2, C4, C6, CP5, CP3, CP1, CPz , CP2, CP4, CP6, P3, POz, P4, POz, O1, O2; reference: FCz, ground: AFz).	32	Bandpass (2–150 Hz) and notch filtering (50 Hz) were applied to the raw EEG signal.	EEGlab.
Shugeng Chen et al., 2020	EEG	The Omega force feedback device w.	Online	Online 31	Filtered by a bandpass filter between 1 and 100 Hz.	SPSS version 23.0 (IBM Inc., Chicago, IL, United States).
Niclas Braun et al., 2017	EEG	mBrainTrain GmbH, Belgrad, Serbia	10–20 systems and positions included 4 Neural plasticity FP1, FP2, F7, F8, FZ, FC1, FC2, T7, C3, CZ, C4, T8, TP9, CP5, CP1, CPz, CP2, CP6, TP10, P3, PZ, P4, O1 and O2. FCz served as reference (CMS) AFz as ground (DRL).	24	Bandpass filtered (8–30 Hz) and segmented from 0.5 to 4.5 s relative to the start of the MI periods.	EEGLab
Belardinelli et al., 2017	EEG	BrainAmp DC amplificador.	System 10–20 (FP1, FP2, F3, Fz, F4, FC5, FC3, FC1, FCz, FC2, FC4, FC6, C5, C3, C1, Cz, C2, C4, C6, CP5, CP3, CP1, CPz , CP2, CP4, CP6, P3, POz, P4, POz, O1, O2; reference: FCz, ground: AFz).	32	Zero-phase bandpass filter (20–500 Hz) with a 3rd order Butterworth filter. Also, a 3rd-order Phase Zero Butterw.	NI

Xin Wang et al., 2018	EEG	USBamp, g.Tec Medical Engineering GmbH, Austria) LADYbird, g.Tec Medical Engineering GmbH, Austria.	System 10–20 (C1, C2, C3, C4, C5, C6, Cz, FC1, FC2, FC3, FC4, FCz, CP1, CP2, CP3, CP4).	16	Bandpass filtering (2–60 Hz) and a notch filtering (48–52 Hz).	SPSS 19 (IBM SPSS, NY, US)
Spychala et al., 2020	EEG	mBrainTrain GmbH, Belgrad, Serbia	10–20 system. Included the following positions: FP1, FP2, F7, F8, FZ, FC1, FC2, T7, C3, CZ, C4, T8, TP9, CP5, CP1, CPz, CP2, CP6, TP10, P3, PZ, P4, O1 and O2. AFz served as ground (DRL) and FCz as reference (CMS).	24	First bandpass filtered from 8 to 28 Hz and then divided into 1.5 s segments in relation to the start of extension, flexion and rest attempts.	EEGLAB
Kober et al., 2015	EEG	NeXus-10 MKII, Mind Media BV.	NI	10	The floor was located on the right mastoid, the reference was placed on the left mastoid. In addition, an EOG channel was recorded in the left eye.	Brainvision Analyzer software (version 2.01, Brain Products GmbH, Munich, Germany).

Prasad et al., 2010	EEG	BSamp amplifier system by g.tec, Graz, Austria.	Locations C3 and C4 (two electrodes placed 2.5 cm anterior and posterior to C3/C4) based on the international 10/20 system.	2	Filtered between 0.5 and 30 Hz with the 50 Hz notch. Each session consisted of 160 trials with 4-s MI-related EEG data sampled at 500 Hz. The EEG was recorded with a g.BSamp amplifier system from g.tec, Graz, Austria. In addition, an EEG cap with an Ag/AgCl electrode set from Easycap™ was used. BCI software based on MATLAB Simulink	Matlab
Remsik et al., 2019	EEG	BCI2000 software	Using a g.GAMMA cap (F5, C5, FC1, C3, P5, F6, C6, P6, Pz, P4, P3, FC2, Cz, CP2, C4, CP1).	16	60 Hz<br>Digital filtering with 4 Hz high pass filter cut and 30 Hz low pass filter cut.	Matlab

Wu Q et al., 2020	EEG fMRI	EEG - LADYbird, g.Tec Medical Engineering GmbH, Schiedlberg,Áustria Fmri - GE 3.0T MRi scanners (DiscoveryTM MR750; GE Healthcare Life Sciences, Chicago, IL, EUA).	International 10–20 system (FC3, FC4, C3, C4, CP3, CP4, C1, C2). EEG signals from electrodes C3 and C4 were used for BCI control - offline analysis (left hemisphere: FC3, C3 and CP3; right hemisphere: FC4, C4 and CP4). FC3/FC4 covers the premotor cortex, while C3/C4 covers the primary motor cortex. CP3/CP4 corresponded to the supramarginal gyrus, which is part of the somatosensory associative cortex.	8	P bandpass filter (2–60 Hz) and a notch filter (48–52 Hz).	SPSS Statistics for Windows, version 20.0. (IBM Corporation, Armonk, NY, USA).
Xiaokang Shu et al., 2018	EEG	GE - BrainAmp amplifier (Brain Products, Gilching, Germany)<br>GC - SynAmps2 system (NeuroScan, U.S.A).	The electrodes were placed according to the extension 10–20 system. The earth channel was located on the forehead, and the reference channel was located at the vertex.	GE – 32<br>GC – 64	GE - Low pass filter setting was 0 to 100 Hz<br>GC - were filtered with an analog bandpass filter from 0.5 to 70 Hz and a notch filter from 50 Hz.	EEGLAB.

Lau CCY et al., 2021	EEG fMRI	LADYbird,g.Tec Medical Engineering, GmbH, Austria).	Located in C1, C2, C3, C4, C5, C6, Cz, FC1, FC2, FC3, FC4, FCz, CP1, CP2, CP3 and CP4 according to the international system 10–20).	16	Power line noise, a bandpass filter from 2 to 60 Hz and 48 to 52 Hz.	SPSS 25.0 (IBM SPSS Statistics, NY, US.
Miao Y et al., 2020	EEG	RecoveriX system (g.tec medical engineering GmbH, Austria)	FC3, FCz, FC4, C5, C3, C1, Cz, C2, C4, C6, CP3, CP1, CPz, CP2, CP4, e Pz)	16	The bandpass filter was set to 0.1-30 Hz.	NI
Choi I et al., 2020	EEG	USBamp biosignal amplifiers (g.tec medical engineering GmbH, Austria	AFz, F7, F3, Fz, F4, F8, FC5, FC3, FC1, FC2, FC4, FC6, T7, C5, C3, Cz, C4, C6, T8, TP7, CP5, CP3, CPz, CP4, CP6, TP8, P3, P4, PO3, PO4, O1, e O2)	32	60 Hz notch filter to remove utility hum in the United States with the BCI2000 system.	JMP® (SAS institute Inc., Cary, NC, USA)
Mrachacz et al., 2016	EEG	EEG amplifier (Nicolet 1, VIASYS Healthcare	FP1, F3, F4, Fz, Pz, P3, P4, C3, C4, e Cz.	1 – monopolar	Bandpass filtered from 0.05 Hz to 10 Hz and subsequently a Laplacian channel (McFarland et al. 1997) was used to increase the MRCP at each epoch.	Matlab
Shindo K et al., 2011	EEG e fMRI	EEG - MOBIlab; Guger Technologies, Graz, Austria) fMRI - 3 T MR scanner (Discover MR750w, GE Medical Systems, United Kingdom).	The electrodes were placed 20 mm lateral and medial to sites C3 (left) and C4 (right) closest to the hand region in the sensorimotor area according to the	5	bandpass filtered (0.5–100 Hz)	MATLAB (MathWorks, United States)

			international system 10-20.			
INSOMNIA						
Hammer BU et al., 2011	EEG	Atlantis amplifier and MiniQ devices by BrainMaster Technologies, Inc. (Bedford, OH).	Cz e C4 (255	19	Baseline filtered delta (1–4 Hz) and beta (12–25 Hz) waves, but not high beta (25–30 Hz).	NI
Schabus M et al., 2017	EEG	Sistema Eldith THERA PRAX (neuroConn)	Fp1, Fpz, Fp2, F3, Fz, F4, F8, T3, C3, Cz, C4, T4, T5, P3, Pz, P4, T6, O1, Oz, O2 plus mastoids A1 and A2 for further offline review - referencing), a horizontal bipolar electrooculogram (HEOG) and a vertical bipolar electrooculogram (VEOG), a bipolar electrocardiogram (ECG) channel, a bipolar electromyogram (EMG) channel, and a respiratory channel (chest wall movements) .	22	They were band-filtered between 0.5 and 70 Hz and a 50 Hz notch filter was applied.	(IBM SPSS Statistics, Version 23; SPSS Inc., Chicago, Illinois)

Fibromyalgia						
Kayiran S et al., 2010	EEG	Alien Technik 3/32 and BrainFeedback-3 configuration	C4 (according to the standard 10–20 system) with 46 Hz bandwidth and the reference electrode placed on the left and the ground electrode on the right earlobe	1	Bandwidth filtered to extract delta (1–4 Hz), theta (4–7 Hz), alpha (8–12 Hz), SMR (12–15 Hz), beta1 (15–20 Hz) and ''high beta'' (22–30 Hz)	Statistical Package for Social Sciences (SPSS) 13.0
Caro XJ et al., 2011	EEG	EEG-BF System (Neurocybernetic Software Package; EEG Spectrum International, Encino	Cz	1	NI	NI
Terrasa JL et al., 2020	EEG fMRI	EEG - QuickAmp Amplifier (Brain Products GmbH, Munich, Germany) a Tesla 3.0 Scanner (SIEMENS MAGNETOM TrioTim syngo MR).	10-20 International System referenced to FCz. The ground electrode was located in the AFz position.	64	High-pass and low-pass filter at 0.10 and 70 Hz, respectively. A 50 Hz notch filter was also applied.	IBM SPSS Statistics v21.
Wu YL et al., 2021	EEG	ProComp Infiniti biofeedback device (Though Technology Ltd., Toronto, Canada)	C3, C4, e Cz	3?	NI	NI
Spinal Cord Injury						

Müller-Putz GR et al., 2014	EEG	GE a portable amplifier (g.tec, Graz, Austria) Neuroscan GC Amplifier	C3, Cz e C4	GE 15<br>GC 32	GE - bandpass filtered between 0.5 and 100 Hz.<br>GC - 50 Hz notch filter<br>.	NI
Vučković A et al., 2019	EEG	Epoch EEG (Emotiv, Estados Unidos)	Placed approximately between C2 and C4	1	Filtered into four frequency bands: wideband 2–30 Hz, 2 theta (4–8 Hz), α alpha (9–12 Hz) and upper β beta (20–30 Hz) using a 5th order Butterworth filter	NI
Mild Cognitive Impairment						
Marlats F et al., 2020	EEG	EEGDigitrack SimplEEG32, Inc. Elmiko Medical sp.z.o.o Limited.	Fp1, Fp2, F7, F3, Fz, F4, F8, T3, C3, Cz, C4, T4, T5, P3, Pz, P4, T6, O1 and O2 w	19	Bandpass filter (0.01–100 Hz).	Statistical software R version 3.3.2 (R Foundation for Statistical Computing).
Amputation of lower limbs						

Takeuchi N et al., 2015	EEG	Neurofax EEG-1000; Cooperação Nihon Kohden, Tóquio, Japão	Placed 1.5 cm anterior and 1.5 cm posterior to C3 (C4 in a left-handed patient) and a pair of Ag-AgCl electrodes placed between Cz and FCz according to the international 10/20 system.	2	Filtered between 0.53 and 30 Hz by a biosignal acquisition system (Neurofax EEG-1000; Nihon Kohden Cooperation, Tokyo, Japan).	MATLAB (The Mathworks Inc).
Quadriplegia and Paraplegia						
Onose G *et al.,* 2012	EEG	BrainAmp128DC, Munich, Germany.	Distributed in a standard limit of 128 channels, in an extended system of 10 to 20.	54	NI	MATLAB (The Mathworks Inc).
Hasan MA et al., 2021	EEG	USB Amplifier Device (Guger Technology, Austria).	Cz, C3, C4, C7, C8, Fz, F3, F4, CP3, CP4, Pz, P3, P4, Oz, O1 e O2.	16	A 5th order high pass Butterworth filter (set to 2 Hz) was applied on the raw EEG data to remove low frequency artifacts while line noise is removed by applying the 48–52 Hz bandstop filter.	sLORETA

Hassan MA et al., 2015	EEG	Usbamp, (Guger technologies, Austria).	F3, Fz, F4, T7, C3, Cz, C4, T8, Cp3, CPz, C4, P4, P3, O1, Oz e O2)	16	Filtered (5th order IIR Butterworth) on selected bands and then squared and smoothed/averaged in a half-second sliding window, updated after each sample, to obtain band power features	Low Resolution Standardized Electromagnetic Tomography sLORETA.
Multiple sclerosis						
Pinter D et al., 2021	EEG fMRI	NeXus-10 MKII, Mind Media B.V.	NI	10	NI?	IBM SPSS Statistics
Kober SE et al., 2019	EEG	NeXus EEG HeadSet, Mind Media B.V.	Cz e C4	1	NI	Brain Vision Analyzer software (version 2.01, Brain Products GmbH, Munich, Germany)
Attention Deficit Hyperactivity Disorder						
Cowley B et al., 2016	EEG	Ambulatory device Enobio Neuroelectrics SL, Barcelona	C4	1	NI	NI
Veilahti AVP et al., 2021	EEG	Ambulatory EEG Amplifier Enobio Neuroelectrics SL, Barcelona	NI	NI	NI	STATA versão 14.2

TABLE 3 - Presentation of the articles included in the meta-analysis.

Author (date)	*Pathology*	*Study design*	*Training dose*	*Sessions (N)*	*Scale*
Rayegani et al., 2014	*Stroke*	*RCT*	*10 OT sessions (5 sessions per week for 2 weeks, 60 min); TONF and TOBF groups received neurofeedback or EMG-BF therapies at the end of each occupational therapy session 3x a week for 4 weeks*	*10*	*Jebsen Test (JHFT)*
Chen et al., 2020	*Stroke*	*RCT*	*10 OT sessions (5 sessions per week for 2 weeks, 60 min); TONF and TOBF groups received neurofeedback or EMG-BF therapies at the end of each occupational therapy session 3x a week for 4 weeks*	*12*	*FMA*
Li M et al., 2013	*Stroke*	*RCT*	*3x a week 1 to 1h and 5 min session for 8 weeks*	*24*	*FMA*
Pichiorri et al., 2015	*Stroke*	*RCT*	*1 month of training:*	*12*	*FMA*
Wang et al., 2018	*Stroke*	*RCT*	*3-5 x per week completed between 5-7 weeks*	*20*	*FMA*
Remsik et al., 2019	*Stroke*	*Cross Over*	*2 to 3 sessions per week 2 hours each*	*9-15*	*ARAT*
Wu et al., 2020	*Stroke*	*RCT*	*4 weeks 5 days a week 2h GC and 1h GE*	*20*	*FMA*
Miao et al., 2020	*Stroke*	*RCT*	*3 x a week for 4 weeks*	*12*	*FMA*
Mrachacz et al., 2016	*Stroke*	*RCT*	*NI*	*3*	*FMA*
Kayiran et al., 2010	*FMG*	*RCT*	*24 week intervention NFB vs Escitalopram 10mg*	*20*	*VAS-pain*
Wu et al., 2021	*FMG*	*RCT*	*8 weeks*	*20*	*FIQR*

Caption: Stroke: Cerebral Vascular Accident; FMG: Fibromyalgia; FIQR Fibromyalgia Impact Questionnaire; FMA: Fugl-Meyer Assessment; ARAT: Action Research Arm Test; VAS-pain: Visual analogue scale.
